# MiR26a reverses enzalutamide resistance in a bone-tumor targeted system with an enhanced effect on bone metastatic CRPC

**DOI:** 10.1186/s12951-024-02438-z

**Published:** 2024-04-02

**Authors:** Yuanyuan Wang, Jiyuan Chen, Luyao Gong, Yunxia Wang, Aino Siltari, Yan-Ru Lou, Teemu J. Murtola, Shen Gao, Yuan Gao

**Affiliations:** 1 School of Pharmacy, Fudan University, Shanghai, 201206 China; 2 Department of Pharmacy, Shanghai Ninth People’s Hospital, Shanghai Jiao Tong University School of Medicine, Shanghai, 200011 China; 3 Faculty of Medicine and Health Technology, Tampere University, Tampere, 33100 Finland; 4 Department of Urology, TAYS Cancer Center, Tampere University Hospital, Tampere, 33100 Finland; 5 Department of Pharmacy, Changhai Hospital, Naval Medical University, Shanghai, 200433 China

**Keywords:** Enzalutamide, Drug resistance, Nanoparticles, Castration-resistant prostate cancer (CRPC), Bone metastatic CRPC

## Abstract

Resistance to androgen receptor (AR) inhibitors, including enzalutamide (Enz), as well as bone metastasis, are major challenges for castration-resistant prostate cancer (CRPC) treatment. In this study, we identified that miR26a can restore Enz sensitivity and inhibit bone metastatic CRPC. To achieve the highest combination effect of miR26a and Enz, we developed a cancer-targeted nano-system (Bm@PT/Enz-miR26a) using bone marrow mesenchymal stem cell (BMSC) membrane and T140 peptide to co-deliver Enz and miR26a. The *in vitro/in vivo* results demonstrated that miR26a can reverse Enz resistance and synergistically shrink tumor growth, invasion, and metastasis (especially secondary metastasis) in both subcutaneous and bone metastatic CRPC mouse models. We also found that the EZH2/SFRP1/WNT5A axis may be involved in this role. These findings open new avenues for treating bone metastatic and Enz-resistant CRPC.

**Supplementary Information:**

The online version contains supplementary material available at 10.1186/s12951-024-02438-z.

## Introduction

Prostate cancer (PCa) is the most prevalent malignancy in men worldwide [1]. Although androgen deprivation therapy (ADT) is initially effective against advanced PCa, it eventually develops into castration-resistant prostate cancer (CRPC) and further progresses into metastatic CRPC (mCRPC) [2]. More than 90% of patients with mCRPC develop bone metastasis (BmCRPC), resulting in a poor prognosis and a survival period of less than two years [3]. Additionally, BmCRPC is frequently associated with skeletal-related events (SREs), such as pathologic fracture secondary to bone metastasis and spinal cord compression [4]. Enzalutamide (Enz), a second-generation androgen receptor (AR) inhibitor, has been approved by the FDA for frontline treatment of mCRPC. Unfortunately, patients treated with Enz treatment ultimately develop resistance *via* various complicated mechanisms [5, 6]. Thus, the development of new strategies to reverse Enz resistance and inhibit BmCRPC is urgently needed.

The histone methyltransferase enhancer of zeste homolog 2 (*EZH2*) was reported to the most highly upregulated gene in CRPC relative to localized PCa, which acts as a transcriptional activator that directly induces *AR* gene expression in a polycomb- and methylation-independent manner [7, 8]. Recently, EZH2 was also reported to mediate epigenomic reprogramming and stemness to facilitate secondary metastasis from bone to other organs and endocrine therapy resistance in breast cancer [9, 10]. However, whether EZH2 contributes to the progression of Enz resistance, BmCRPC, and secondary metastasis to other organs is still unknown. Moreover, noncanonical WNT signaling is considered a major contribution to PCa progression in CRPC patients. WNT5A promotes BmCRPC by regulating CCL2 and BMP6 [11]. Elevated WNT5A expression also has been demonstrated in patients resistant to Enz treatment [12, 13]. Whether there is interaction between EZH2 and WNT5A in PCa remains to be thoroughly investigated. Our bioinformatics analysis results also indicated high expressions of EZH2 and WNT5A in Enz-resistant BmCRPC patients. Thus, targeting EZH2 or WNT5A may bring potential therapy for Enz resistance and BmCRPC.

Based on the TargetScan database, we identified that miR26a could target WNT5A and EZH2 directly. MiR26a has been demonstrated to be downregulated in PCa tissue and regulates extracellular vesicle secretion to suppress PCa progression [14]. Due to the critical regulatory roles of miR26a, we hypothesized that miR26a could reverse Enz resistance and inhibit BmCRPC. The combination of miR26a with Enz might provide an enhanced clinical therapy effect for CRPC.

**Scheme 1:**
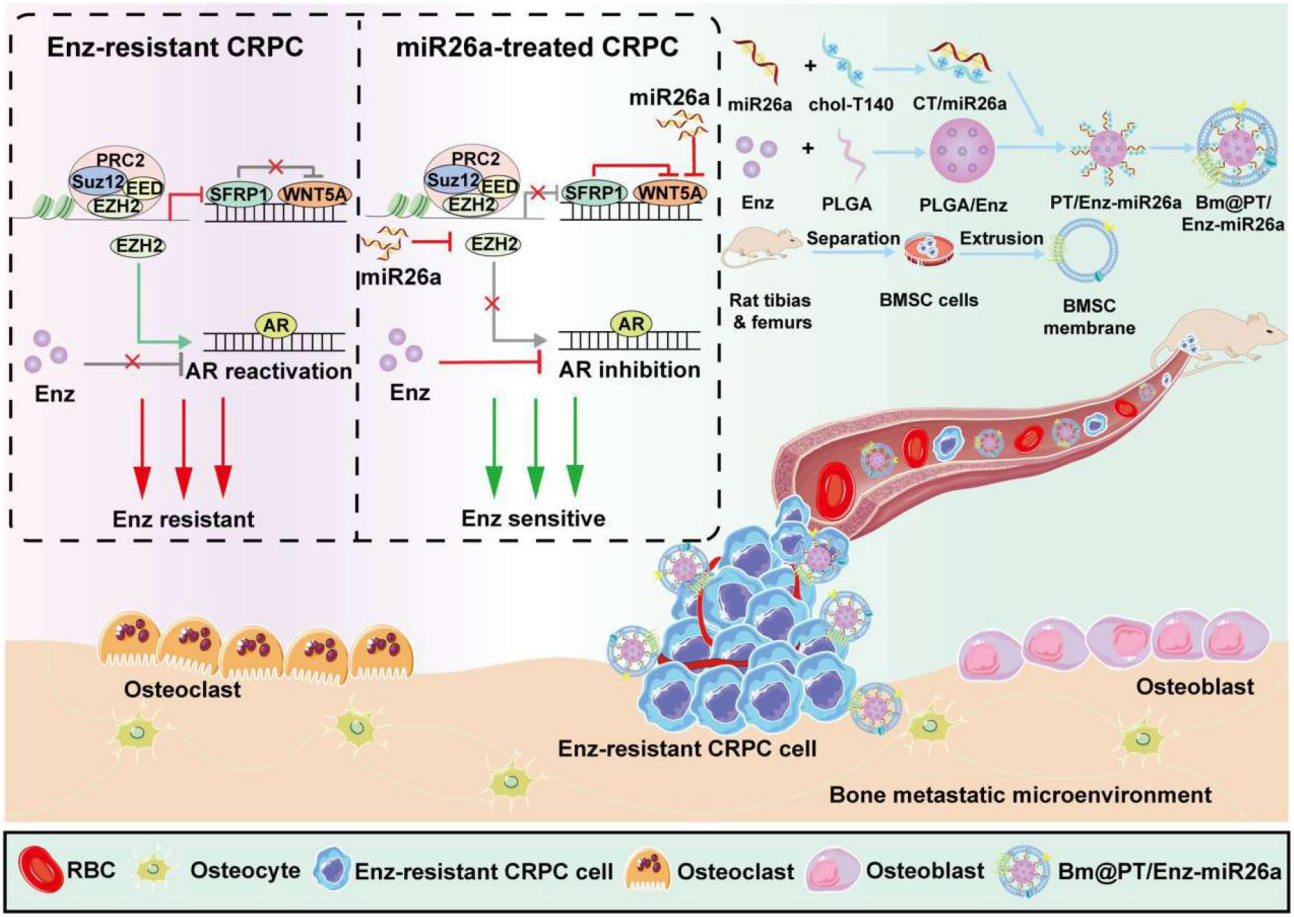
Schematic illustrations of the construction and mechanism of action of Bm@PT/Enz-miR26a in Bone-tumor microenvironment

To co-deliver Enz and miR26a to tumor and bone metastatic sites, we constructed a core-shell-like biomimetic bone-tumor targeted nano-system. Enz is difficult to administer intravenously due to its high daily dosage and hydrophobic property [15]. Here, we selected an FDA-approved biodegradable polymer poly (lactic-co-glycolic acid) (PLGA) to improve Enz’s drug loading [16]. For miR26a delivery, cationic peptide T140 was used with its N-terminus modified by cholesterol to generate an amphiphilic peptide (CT), which could self-assemble and form a micelle-like structure in aqueous solutions. Previous studies have showed that MSC membranes exhibit priority targeting to inflammation, injury sites, tumors, and especially bone marrow. The inflammatory migration property and recognition sites for the interaction between MSCs and tumor cells make them ideal candidates for targeted biomaterials [17, 18]. Our former research demonstrated that bone marrow mesenchymal stem cell (BMSC) membranes exhibited an excellent ability to target bone metastatic PCa since BMSC membranes retain the entire set of receptor ligands on the surface of BMSCs [19]. Therefore, the nano-system was coated with BMSC membranes to form a bone-tumor targeting biomimetic drug-loaded nano-system (Bm@PT/Enz-miR26a). We hypothesized that Bm@PT/Enz-miR26a could overcome Enz resistance, halt the development of additional metastasis, and prevent the proliferation of established lesions within bones (Scheme 1).

## Materials and methods

### Materials

Poly (lactic-co-glycolic acid) (PLGA) (75:25, 15 kDa) was purchased from Dongguan Haosheng Plastic Materials Co., Ltd. Enzalutamide(Enz)was generously provided by Shanghai Fosun Pharmaceutical Group Co., Ltd. The synthesis and purification of cholesterol-modified T140 peptide was entrusted to Shanghai GL Biochem Co., Ltd. Hsa-miRNA-26a-5p mimics (sense: 5’-UUCAAGUAAUCCAGGAUAGGCU-3’, antisense: 5’-CCUAUCCUGGAUUACUUGAAUU-3’), scramble miRNA (negative control, NC), FAM-labeled miRNA (miFAM), siSFRP1 (sense: 5’-CAUCAUUGAACAUCUCUGUTT-3’, antisense: 5’-ACAGAGAUGUUCAAUGAUGTT-3’), siEZH2 (sense: 5’- GCUCCUCUAACCAUGUUUATT-3’, antisense: 5’- UAAACAUGGUUAGAGGAGCTT-3’), siNC1, and siNC2 were purchased from Shanghai GenePharma Co., Ltd. Dichloromethane (DCM) and dimethylsulfoxide (DMSO) were purchased from Aladdin. Polyvinyl alcohol (PVA) was purchased from Sigma‒Aldrich.1,1-Dioctadecyl-3,3,3’,3’-tetramethylindotricarbocyanine iodide (DiR, λex/λem = 750/780 nm) was purchased from Shanghai Bioscience Technology Co., Ltd. 1,1’-Dioctadecyl-3,3,3’,3’-tetramethylindodicarbocyanine, 4-chlorobenzenesulfonate salt (DiD, λex/λem = 644/655 nm), 3,3’-dioctadecyloxacarbocyanine perchlorate (DiO, λex/λem = 484/501 nm), and 2-(4-amidinophenyl)-6-indolecarbamidine dihydrochloride (DAPI, λex/λem = 340/488 nm) were purchased from Beyotime Biotechnology. 4% paraformaldehyde fixative and phosphate buffered saline (PBS) were purchased from Wuhan Service bio Technology Co., Ltd. Dulbecco’s modified Eagle’s medium (DMEM), high-glucose medium, Roswell Park Memorial Institute (RPMI) 1640 medium, fetal bovine serum (FBS), and 0.25% trypsin were purchased from Gibco. DMEM/nutrient mixture F-12 (DMEM/F12) was purchased from HyClone. Keratinocyte medium was purchased from Shanghai Zhong Qiao Xin Zhou Biotechnology Co., Ltd. EZH2 Ab (rabbit, Cat.#AF5150, Lot.#00095479, Affinity), H2K27me3 Ab (rabbit, Cat.#DF6941, Lot.#46k3813, Affinity), SFRP1 Ab (rabbit, Cat.#DF01172, Lot.#35u4433, Affinity), WNT5A Ab (rabbit, Cat.#BM5137, Lot.#BST17695137, Boster), AR Ab (rabbit, Cat.#AF6137, Lot.#84f0092, Affinity) and GAPDH Ab (mouse, Cat.#ab8245, Abcam) were purchased for Western blotting study.

### Cell culture

The human prostate cell line RWPE-1, the human prostate cancer cell lines C4-2B, the human osteosarcoma cell line MG-63, and the HEK-293T cell line were purchased from the Cell Bank of the Chinese Academy of Science (CAS) (Shanghai, China). CAS uses short tandem repeat (STR) profiling to test and authenticate cell lines. C4-2B cells were chronically exposed to RPMI 1640 medium (10% FBS, 1% penicillin/streptomycin) containing Enz (20 µM) for 6 months. C4-2B cells resistant to Enz are referred to as C4-2B EnzR. RWPE-1 cells were maintained in keratinocyte medium supplemented with 1% keratinocyte growth factor and 1% penicillin/streptomycin. C4-2B, MG-63, and HEK-293T cells were maintained in DMEM supplemented with 10% FBS and 1% penicillin/streptomycin. C4-2B EnzR cells were maintained in Enz-containing RPMI 1640 medium supplemented with 10% FBS and 1% penicillin/streptomycin. For the Transwell co-culture assay (6-well plate, 0.4-µm pore size, 3450, Corning), C4-2B EnzR cells were seeded in the lower chamber at a density of 5 × 10^3^ cells per well, while MG-63 cells were seeded in the upper chamber at the same density. All cells were cultured in a humidified environment at 37 °C in a 5% CO_2_ atmosphere, and passages 10 to 15 of these cell lines were used in this study.

### Animals

Healthy BALB/c male nude mice (4 weeks, 15–18 g) and Sprague‒Dawley male rats (SD, 4 weeks, 80–120 g) were obtained from Shanghai Laboratory Animal Center (Chinese Academy of Sciences, Shanghai, China). All experimental animals were contained in a clean animal room under specific pathogen-free (SPF) conditions. All animal experiments were approved by the laboratory animal ethics committee of Fudan University, and all animals were anesthetized or sacrificed with CO_2_.

### Bioinformatics analysis

The raw expression profile was obtained from the Gene Expression Omnibus (GEO; https://www.ncbi.nlm.nih.gov/geo/query/acc.cgi?%20acc=GSE32269) to compare gene expression levels between metastatic PCa (CRPC) tissues and primary PCa tissues. The GSE32269 dataset contained 22 primary PCa samples and 29 metastatic PCa (CRPC) samples. The differentially expressed genes (DEGs) were detected using the ‘Limma’ package of R software, with cut-off criteria of a false discovery rate (FDR) < 0.05 and absolute |logFC|>1. Then, a volcano plot was drawn.

### Quantitative real-time polymerase chain reaction (qPCR)

C4-2B EnzR cells were seeded in 6-well plates at 2 × 10^4^ cells per well overnight and then transfected with Lipofectamine/miR26a mimics (sense: UUCAAGUAAUCCAGGAUAGGCU) and Lipofectamine/miR26a inhibitors (sense: AGCCUAUCCUGGAUUACUUGAA) for 48 h. Reverse transcription PCR was carried out using a miRNA 1st Strand cDNA Synthesis Kit (by stem‒loop) (miR26a primer: GTCGTATCCAGTGCAGGGTCCGAGGTATTCGCAC). The cDNA was used to amplify the miR26a using miRNA Universal SYBR qPCR Master Mix. MiR26a was normalized to the housekeeping gene *GAPDH*.

### Western blotting

Total cell lysates were collected in RIPA buffer. The protein concentration was determined with a BCA assay kit. After that, the protein solution was added to the reduced protein loading buffer at a ratio of 4:1 and denatured in a boiling water bath for 15 min. Then, the protein was separated by SDS-polyacrylamide gel electrophoresis and transferred to a polyvinylidene difluoride membrane. The transferred membrane was placed into the TBST incubation tank for a quick wash and blocked for 30 min at room temperature with a blocking buffer. After incubating with primary antibody (EZH2: Affinity AF5150; H3K27me3: Affinity DF6941; SFRP1: Affinity DF10172; WNT5A: Boster BM5137; AR: Affinity AF6137; GAPDH: Abcam ab8245) and conjugated secondary antibody (HRP-conjugated affinipure Goat Anti- Rabbit lgG (H + L), Proteintech SA00001-2; HRP-conjugated Affinipure Goat Anti-Mouse IgG (H + L), Proteintech SA00001-1), the membrane was imaged in a gel imager. PageRuler prestained protein (26,616) was purchased from ThermoFisher.

### Isolation of rat BMSC cells and cell membrane extrusion

Rat BMSCs were isolated, and the cell membrane was extruded as reported [32]. In detail, BMSCs were extracted from the tibias and femurs of 4-week-old SD male rats with a lymphocyte separation kit. At passages 3 to 6, BMSCs were collected to extract cell membranes. BMSCs were continuously squeezed back and forth with a micro extruder (25 mm) (Changsha Nayi Instrument Technology Co., Ltd., China) 21 times and sonicated for 5 min (Kunshan Ultrasonic Instruments Co., Ltd., KQ-500E ultrasonic cleaner, China). The BMSC cell membrane was extracted and purified by gradient centrifugation at 2,000 g, 10,000 g, and 10,000 g. The zeta potential was detected by the dynamic light scattering (DLS) system (Malvern), and the total membrane protein was evaluated by the BCA method. The BMSC cell membrane was stored at -80 °C for subsequent experiments.

### Preparation of Bm@PT/Enz-miR26a nanoparticles

The emulsification-solvent evaporation method was utilized to synthesize nanoparticle cores. Briefly, PLGA and Enz were first dissolved in DCM, and then the organic phase was added to an aqueous phase containing 1% PVA. The mixture was emulsified by using an ultrasonic probe (YM-650Y, Shanghai Yuming Automation & Technology Co., Ltd.) in an ice bath for 5 min. The suspension mixture was stirred continuously overnight at RT. The Enz-loaded nanoparticles were separated by centrifugation at 4 °C and 14,000 × g for 30 min. Cholesterol-modified CT peptides were incubated with miR26a for 30 min at RT. After that, Enz-loaded PLGA nanoparticles were incubated with CT-miR26a for 30 min *via* electrostatic adsorption. The BMSC membranes were subsequently coated onto the nanoparticle cores through sonication at a power of 500 W for 5 min. The Enz-loaded nanoparticles were separated by centrifugation at 25 °C and 10,000 rpm for 1 h.

### Gene transfection assay

HEK-293T cells were seeded in 48-well plates at 3 × 104 cells per well in 0.5 mL DMEM for the gene transfection assay. Then, CT/pEGFP (Beyotime, D2707) was prepared with different N/P ratios of CT peptide and plasmid of enhanced green fluorescent protein (pEGFP), while PEI/pEGFP (PEI, MW25000, Yeasen Biotechnology, 40815ES03) was used as a control group. After incubating with HEK-293T cells for 4 h, the medium was replaced with fresh DMEM, and plates were incubated for an additional 44 h. The images were observed under a fluorescence microscope, and EGFP-positive cells were calculated using ImageJ software.

### Drug loading content and encapsulation efficiency

The concentration of Enz in Bm@PT/Enz-miR26 was determined by HPLC-UV under the following conditions: Eclipse plus C18 column (4.6 × 250 mm, 5 μm), 0.05% trifluoroacetic acid and acetonitrile (70:30, v/v) as the mobile phase, flow rate (1 mL min^− 1^), column temperature at 30 °C, an injection volume of 10 µL, and a wavelength of 220 nm. Briefly, Bm@PT/Enz-miR26a (1 mL) solution was diluted 10 times with acetonitrile and then demulsified in an ultrasonic cleaner for 10 min. Additionally, Enz (4 mg) was diluted with acetonitrile up to 20 mL as the reference solution. The encapsulating efficiency (EE%) and drug loading (DL%) were calculated as follows:

$$\text{EE}\%=\frac{\text{Mass}\;\text{of}\;\text{Enz}\;\text{in}\; \text{the}\; \text{nanoparticle}}{\text{Total}\;\text{mass}\;\text{of}\;\text{Enz}}\times 100\%$$

$$\text{DL}\%=\frac{\text{Mass}\;\text{of}\;\text{Enz}\;\text{in}\; \text{the}\;\text{nanoparticle}}{\text{Total}\;\text{mass}\;\text{of}\;\text{nanoparticle}}\times 100\%$$

### Characterization of Bm@PT nanoparticles

The particle size, PDI, and zeta potentials were detected using a DLS system. The morphology of the biomimetic nanoparticles was observed by transmission electron microscopy (TEM, FEI TECNAI G2 S-TWIN, USA). Moreover, Bm@PT nanoparticles were adjusted to 1× PBS or 0.5× FBS at RT, and the stability was measured once a day for 7 consecutive days. To track nanoparticles, DiD (2 µg) dye was added to the PLGA DCM solution prior to synthesis. Then, PT was coated with DiO-stained BMSC cell membranes, while the nuclei of C4-2B EnzR cells were stained with DAPI. A High-Content Analysis System (Operetta CLS, PE) was utilized to observe the colocalization of the BMSC cell membranes and polymeric cores after internalization by C4-2B EnzR cells.

### In vitro cell uptake study

To evaluate the cell uptake efficiency, C4-2B EnzR cells were seeded in 12-well plates at a concentration of 2 × 10^5^ cells/well overnight. Nile Red (Sigma‒Aldrich) and miFAM were chosen as model drugs for co-incubation with each group in serum-free medium for 4 h. Flow cytometry was performed to examine nanoparticle uptake and detect the fluorescence intensity of each group. All flow cytometry tests were conducted on a FACSCanto II flow cytometer (BD Biosciences), and the data were analyzed using FlowJo software.

### Lysosome escape assay

To investigate the ability of lysosomes to escape, C4-2B EnzR cells were seeded in 24-well plates at a concentration of 1 × 10^5^ cells/well overnight. Coumarin-6 was encapsulated in Bm@PT (Bm@PT/C6, 20 ng·mL^− 1^) for 30 min. LysoTracker Red (50 ng·mL^− 1^) was used to label lysosomes in C4-2B EnzR cells, and nuclei were stained with DAPI. After 1 h and 4 h of incubation, the images were obtained by a High-Content Analysis System.

### In vitro cytotoxicity evaluation

C4-2B and C4-2B EnzR cells were seeded in 96-well plates at a density of 8 × 10^3^ cells·mL^− 1^ and cultured overnight. Then, cells were added to various concentrations of formulations and cocultured for 48 h. Cell viability was assessed by a Cell Counting Kit-8 (Beyotime) based on a microplate reader (Synergy H1, BioTek). IC_50_ values were calculated by GraphPad Prism 8.0 software *via* the equation of ‘[Inhibitor vs. normalized response – Variable slope]’.

### Cell cycle arrest and apoptosis assays

To evaluate the effects of Enz and miR26a on cell cycle arrest, C4-2B EnzR cells were seeded in 6-well plates at a density of 3 × 10^5^ cells·mL^− 1^ and cultured overnight. Then, different formulations were added to cells (Enz: 25 µg·mL^− 1^, miR26a: 0.2·µg mL^− 1^) and cocultured for 48 h. A Cell Cycle Staining Kit (Multi Sciences) was used to detect the influence of cell cycle arrest. For the cell apoptosis assay, C4-2B EnzR cells were seeded in 12-well plates at a density of 2 × 10^5^ cells·mL^− 1^ and cultured overnight. The Annexin V-FITC/PI apoptosis kit (Multi Sciences) was used to detect the apoptosis of C4-2B EnzR cells with a concentration of 30 µg·mL^− 1^ Enz and 0.25 µg·mL^− 1^ miR26a. All flow cytometry analyses were based on a FACSCanto II flow cytometer (BD Biosciences), and the data were analyzed using FlowJo software.

### In vitro cell migration and invasion assays

Before the assays, C4-2B EnzR cells were cultivated with non -serum medium for 24 h. Then, C4-2B EnzR cells were seeded on the upper chamber of the Transwell plate (8-µm, Corning) at a density of 10^5^ cells 100^− 1^·µL^− 1^ (without FBS), while DMEM (800 µL) containing 20% FBS was added as a chemokine to the lower chamber. For the anti-invasion assay, Matrigel (BD) was placed in the upper chamber of the Transwell plate. After coincubation with each group (Enz: 50 µg·mL^− 1^, miR26a: 0.5 µg·mL^− 1^) for 24 h (anti-migration assay) and 48 h (anti-invasion assay), the cells on the surface of the upper chamber were carefully wiped off with a sterile cotton swab. The cells were fixed with methanol (TEDIA) for 30 min, stained with 0.1% crystal violet (Beyotime) for 20 min, and washed 3 times with PBS. Nine fields were randomly selected for observation and photographed under the bright-field field of a fluorescence microscope (DP80, Olympus) for subsequent statistical analysis. And the migratory/invasive cells in the images were calculated by Image J software.

### Formation and characterization of three-dimensional (3D) multicellular tumor spheroids

*In* vitro 3D multicellular tumor spheroids of C4-2B EnzR cells and MG-63 cells were developed by a liquid-overlay method. Briefly, sterile agarose (AR, Biowest) was added to 96-well plates (50 µL·well^− 1^). Subsequently, a total of 2 × 10^4^ DiO-stained C4-2B EnzR cells and DiD-stained MG-63 cells were seeded in 96-well plates at a ratio of 1:1 and centrifuged at 1,000 g for 10 min at 4°C. During spheroid formation, DMEM was replaced every 2 days. The tumor spheroids were grown up to 500 μm and measured under a confocal laser scanning microscope (CLSM, Carl Zeiss LSM710, Zeiss).

### Penetration of Bm@PT nanoparticles in 3D multicellular tumor spheroids

The tumor spheroids were prepared without dye and developed as previously described. Bm@PT was loaded with DiR (Bm@PT/DiR) and coincubated with tumor spheroids for 8 h. Then, the tumor spheroids were washed three times with ice-cold PBS and fixed with 4% paraformaldehyde for 30 min. The nuclei of C4-2B EnzR cells were stained with DAPI. Subsequently, the tumor spheroids were transferred into confocal dishes, and images were acquired by CLSM at 10× objective magnification using Z-stack imaging from the bottom of the spheroids at 20 μm intervals. The images were processed with Zen 3.4 software.

### Cell viability in 3D multicellular tumor spheroids

Tumor spheroids with a diameter of approximately 400 μm were coincubated with different formulations for 48 h (Enz: 50 µg·mL^− 1^, miR26a: 1 µg·mL^− 1^). The cytotoxicity was evaluated by a Live/Dead Kit (Beyotime), and all images were captured by CLSM.

### Establishment of subcutaneous CRPC and BmCRPC mouse models

For the subcutaneous CRPC model, 5 × 10^5^ C4-2B EnzR cells were mixed with Matrigel at a ratio of 1:1 and then subcutaneously inoculated into the right hind limb of 6-week-old male BALB/c nude mice. For the BmCRPC mouse model, the femur and tibia of the left hind limb were bent at an angle of 90°, and 5 × 10^5^ cell suspensions were inoculated into the bone marrow cavity along the long axis of the tibia.

### In vivo biodistribution study

The subcutaneous CRPC and BmCRPC mouse models were established as previously described. Free DiR and DiR-loaded Bm/PT nanoparticles were injected *via* the tail vein at a concentration of 10 mg·kg^− 1^. The mice were scanned at 0, 2, 4, 6, 8, 10, 12, and 24 h by an in vivo imaging system (IVIS, Bio-Real Quick View 3000, Bio-Real Sciences) and sacrificed at 24 h. All hearts, livers, spleens, lungs, kidneys, tumors, and left hind limb tibias were collected. The fluorescence of dissected organs was measured by an in vivo imaging system. All data were analyzed by Quick View 3000 software.

### In vivo antitumor effects of the biomimetic nano-system

The subcutaneous CRPC and BmCRPC mouse models were established as conducted above. The mice were randomly grouped (*n* = 5): (a) saline; (b) blank nanoparticles; (c) Enz; (d) miR26a; (e) PT/Enz; (f) PT/miR26a; (g) PT/Enz-miR26a; (h) Bm@PT/Enz-miR26a (Enz: 20 mg kg^− 1^, miR26a: 0.5 mg kg^− 1^). The tumors reached a mean volume of approximately 100 mm^3^, and mice were treated by tail vein injection every 2 days for 2 weeks. The first dose was recorded as the first day, while the tumor volume and body weight were measured every other day. The tumor volume calculation formula was V = L × W^2^/2, in which L is the length of the longest axis of the tumor and W is the length of the axis perpendicular to the longest axis. All the animals were sacrificed 16 days after the first injection. The tumors were dissected and weighed. The tumors and organs were collected for histological examination.

### Survival study

When the tumor volume reached 100 mm^3^, all mice were randomly divided into 8 groups, and different formulations were injected intravenously every 2 days for 2 weeks. The mouse weight and tumor size were measured every 2 days for survival study. Considering the animal ethics, tumor-bearing mice were sacrificed when the tumor volume reached 2000 mm^3^.

### In vivo safety evaluation

The mice were anesthetized to obtain blood samples from the retro-orbital venous plexus, and these samples were obtained to detect biochemical indicators (ALT, AST, BUN, and CR). The excised organs (hearts, livers, spleens, lungs, and kidneys), tumors, and left hind limb tibias were fixed in 4% paraformaldehyde, embedded in paraffin, and cut into 5 μm thick slices. Tissue sections were stained with hematoxylin and eosin (H&E) and observed under an optical microscope.

### In vivo apoptosis of tumor cells

For TUNEL staining, the paraffin sections were dewaxed in water. After repair with proteinase K, the membrane was broken and equilibrated at room temperature for 10 min. Then, the reaction solution was added and incubated in a 37 °C incubator for 2 h. Finally, the nuclei were counterstained with DAPI and incubated for 10 min at room temperature in the dark. Sections were observed under a fluorescence microscope, and images were collected (DAPI emits blue light at an ultraviolet excitation wavelength of 330–380 nm and an emission wavelength of 420 nm; FITC exhibits an excitation wavelength of 465–495 nm and an emission wavelength of 515–555 nm and emits green).

### MicroCT imaging and analysis of bone loss

The left hind limb tibias were collected, and normal right hind limbs were used as controls (*n* = 5). Bone images were obtained using microCT (Siemens Inveon microCT, SD_000_N8-875, Germany) under 80 kVp conditions. A tissue-equivalent phantom (Siemens, Germany) was used to establish a standard line of bone mineral density (BMD), which was used to convert the heat unit (HU) values detected by microCT to the BMD values (mg·cc^− 1^). Five areas of each tibia were randomly selected to measure the HU values and adjusted to the BMD values according to the standard line.

### Statistical analysis

All values are presented as the mean ± standard deviation (SD). All experiments were performed with at least three biologically independent replicates, and all statistical analyses were performed using GraphPad Prism 8.0 software (GraphPad software, CA, USA). Each group was compared by one-way ANOVA with statistical significance set at *p < 0.05, **p < 0.01, ***p < 0.001 and ****p < 0.0001.

## Results

### Bioinformatics prediction and analysis of related genes in CRPC

**Fig. 1:**
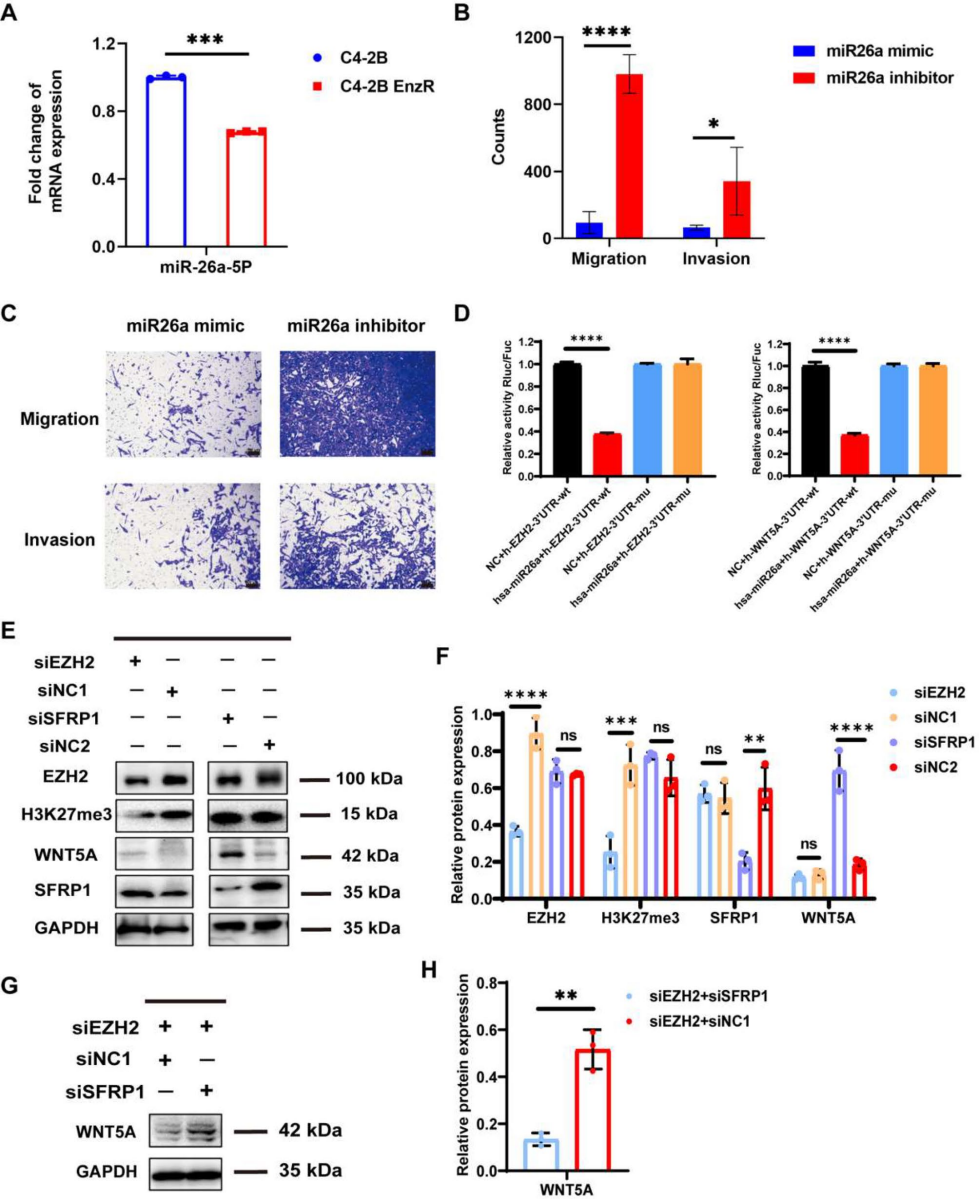
The mechanism of regulatory relationships among EZH2, SFRP1, and WNT5A. **(A)** The miR26a level in C4-2B and C4-2B EnzR cells. *n* = 3, mean ± SD, ***p < 0.001, one-way ANOVA. **(B)** Statistical analysis of each group. *n* = 5, mean ± SD, *p < 0.05, ****p < 0.0001, one-way ANOVA. **(C)** In vitro cell migration and invasion assays. Microscope images of anti-migration and anti-invasion effects in each group (visualized with 0.1% crystal violet, scale bar: 100 μm). C4-2B EnzR cells were incubated with miR26a mimic and miR26a inhibitor, respectively (miR26a mimic/inhibitor: 1 µg·mL^− 1^). **(D)** Dual luciferase reporter assays were used to verify the direct targeting of miR26a on EZH2 and WNT5A, h-EZH2-3’UTR/h-WNT5A-3’UTR: 0.16 µg, hsa-miR-26a-5p/NC: 5 pmol. *n* = 3, mean ± SD, ****p < 0.0001, one-way ANOVA. **(E)** Protein expressions of EZH2, WNT5A, H3K27me3, and SFRP1 were detected *via* western blotting in siEZH2, siSFRP1, and their negative control groups (siNC1: EZH2 negative control, siNC2: SFRP1 negative control). **(F)** Statistical difference of the ratio of gray values. **(G)** Protein expressions of WNT5A were detected *via* western blotting in siEZH2 + siSFRP1 and siEZH2 + siNC1 groups. **(H)** Statistical difference of the ratio of gray values. *n* = 3, mean ± SD, **p < 0.01, ***p < 0.001, ****p < 0.0001, one-way ANOVA

Down-regulation of miR26a was reported in PCa tissue as compared to that in normal prostate tissue [14]. In order to evaluate the miR26a level in Enz-resistant BmCRPC, C4-2B Enz-resistant (C4-2B EnzR) cells were generated *via* chronic culture of C4-2B cells in the complete medium containing 20 µM Enz for 6 months [20] (Fig. S1A). The protein expression of AR and AR variants (e.g., AR-V7) in C4-2B EnzR cells was much higher than that in C4-2B cells Supporting Fig. S1B). Moreover, the proliferation of resistant cells was unaffected in the presence of Enz, while the proliferation of C4-2B cells was significantly inhibited (Supporting Fig. S1C). Consistently, 5 days of continued growth was observed for C4-2B EnzR cells compared with C4-2B cells in Enz-containing media (Supporting Fig. S1D). The above results indicated that Enz-resistant C4-2B EnzR cells were successfully established. Then, we identified that miR26a was downregulated in C4-2B EnzR, as compared to that in C4-2B cells (***p < 0.001) (Fig. 1A). And C4-2B EnzR cells treated with miR26a mimic showed significant inhibition of invasion (*p < 0.05) and migration (****p < 0.0001) compared with its inhibitor (Fig. 1B-C).

The miRNA target prediction algorithm TargetScan predicted that the 3′-UTR of EZH2 and WNT5A mRNA contain a putative miR26a binding site (Supporting Fig. S2A-B). To further investigate the roles of EZH2 and WNT5A in Enz-resistant BmCRPC, bioinformatics analysis of the GEO dataset (GSE32269) was performed. The results showed that the expressions of EZH2, WNT5A, and AR were elevated while secreted frizzled related protein 1 (SFRP1) was downregulated (Supporting Fig. S2C-D). SFRP1 belongs to the SFRP family, which is structurally similar to WNT ligands. Thus, SFRP1 is considered a WNT pathway antagonist, which can regulate signaling by binding to WNT proteins or frizzled receptors [21]. The direct regulation of EZH2 on SFRP1 in osteoarthritis [22] and laryngeal carcinoma [23] has been reported before. However, the relationship of EZH2, SFRP1, as well as WNT5A remains to be explored in Enz-resistant BmCRPC. Thus, PCR results showed that EZH2 and WNT5A were significantly upregulated while SFRP1 was decreased in C4-2B EnzR cells compared to those in C4-2B groups (Supporting Fig. S2E).

### MiR26a directly regulated the expression of EZH2 and WNT5A

To determine the regulatory effect of miR26a on EZH2 and WNT5A, dual luciferase reporter assays were used to verify the target relationship among miR26a, EZH2, and WNT5A. The wild-type reporter plasmid (h-EZH2-3’UTR-wt/h-WNT5A-3’UTR-wt) and the mutant reporter plasmid (h-EZH2-3’UTR-mu/h-WNT5A-3’UTR-mu) were constructed (Supporting Fig. S3A-C). Compared with the NC-treated group, hsa-miR-26a-5p significantly downregulated luciferase expression in h-EZH2-3’UTR-wt cells (****p < 0.0001). After mutation, hsa-miR-26a-5p failed to downregulate the expression of luciferase in the h-EZH2-3’UTR-mu (*p* > 0.05) (Fig. 1D). Similar results were observed in the dual-luciferase reporter assay for WNT5A (Fig. 1D). Together, the above results showed that miR26a directly targeted EZH2 and WNT5A mRNA 3’UTR and downregulated the expression of EZH2 and WNT5A.

### EZH2 downregulation improved SFRP1 expression, and SFRP1 inhibition restored WNT5A expression

As a direct target of EZH2 [24], SFRP1 is also an inhibitor of WNT5A [25, 26]. To investigate the molecular mechanisms and biological functions of EZH2 and SFRP1 in Enz-resistant CRPC cells, the C4-2B EnzR cells were transfected with siEZH2 and siSFRP1 using Lipofectamine. After 48 h of co-incubation, the protein levels of EZH2 and H3K27me3 were markedly decreased in the siEZH2 group as compared with those in the negative control (siNC1) group, while no obvious changes were observed in the siSFRP1 group. Moreover, the SFRP1 expression was higher in the siEZH2 group and lower in the siSFRP1 group than in negative control groups (Fig. 1E). Altogether, these findings indicated that EZH2 could directly downregulate SFRP1 expression. Then, we detected the variation in WNT5A after the inhibition of SFRP1. The western blotting results showed that the expression of WNT5A was upregulated in siSERP1-treated group. Although the expression level of WNT5A did not show statistical difference between siEZH2-treated and siNC1-treated groups, WNT5A expression in siNC1-treated group was numerically higher than in siEZH2-treated group. (Fig. 1F). These results indicated that WNT5A was a downstream regulatory factor of SFRP1 and was directly regulated by SFRP1.

Next, we validated the mechanism through a rescue experiment. SiEZH2 and siSFRP1 were transfected into resistant cells. The protein expression of WNT5A was detected *via* western blotting, which showed that the protein level of WNT5A in the siEZH2 + siSFRP1 group was higher than that in the siEZH2 + siNC1 group (**p < 0.01) (Fig. 1G-H). All these findings showed that the downregulation of SFRP1 reversed the inhibition of EZH2 silencing in Enz-resistant cells by activating WNT5A.

### Biomimetic nano-system realized the combined delivery of Enz and miR26a

**Fig. 2:**
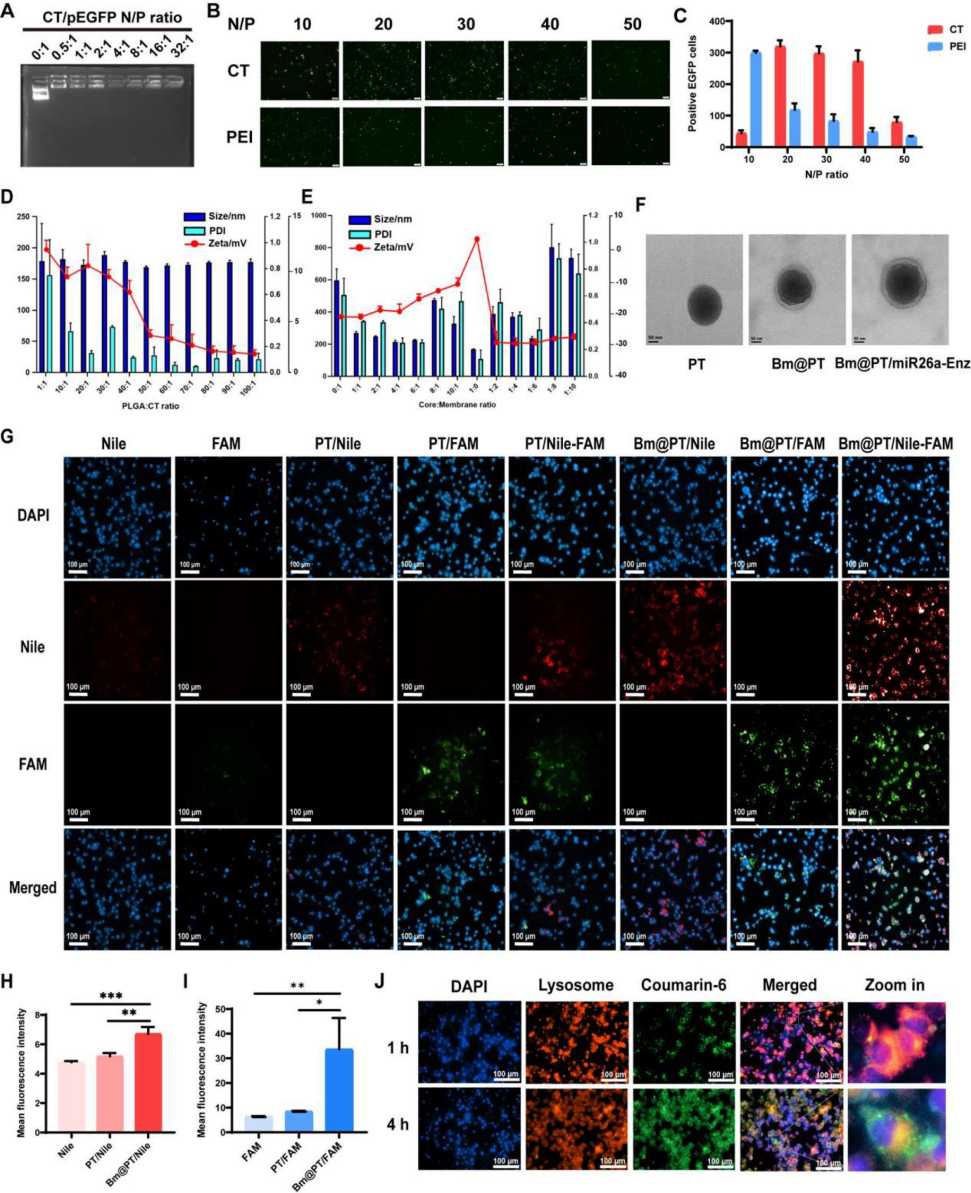
Synthesis and characterization of Bm@PT/Enz-miR26a. **(A)** The agarose gel electrophoresis results of different N/P ratios of CT/pEGFP. **(B)** Microscope images of transfection ability of PEI and CT in HeK-293T cells at different N/P ratios. Scale bar: 100 μm. **(C)** Statistical analysis of the number of EGFP-positive cells. *n* = 3, mean ± SD, ****p < 0.0001, one-way ANOVA. **(D)** Optimum ratio of PLGA to CT. **(E)** Optimum ratio of nanoparticles to BMSC membrane. **(F)** TEM images of PT, Bm@PT, and Bm@PT/Enz-miR26a. Scale bar: 50 nm. **(G)** High-Content Analysis System images of intracellular distribution. Nile/miFAM: 20 ng·mL^− 1^. Scale bar: 100 μm. **(H–I)** Cellular uptake of nanoparticles by flow cytometry. Nile/miFAM: 20 ng·mL^− 1^, *n* = 3, mean ± SD, *p < 0.05, **p < 0.01, ***p < 0.001, one-way ANOVA. **(J)** High-Content Analysis System images of lysosome escape. C6: 20 ng·mL^− 1^, Lysotracker red: 50 ng·mL^− 1^. Scale bar: 100 μm

Based on the regulatory role of miR26a in the EZH2/SFRP1/WNT5A axis, a pathway closed related to Enz resistance, we hypothesized that the combination of miR26a and Enz could restore the sensitivity of resistant cells to Enz. To achieve the combined targeted delivery of Enz and miR26a, we constructed a ‘core-shell’ like biomimetic nano-system. Firstly, T140 peptide (RRXCYRKKPYRXCR), which is rich in arginine and histidine, was modified by cholesterol to condense miR26a (CT/mi26a) (Supporting Fig. S4A). The chromatographic peak of CT has a retention time of 7.734 min and its molecular weight is 2449.07, with the purity of 96.7% (Supporting Fig. S4B-C). Then, PLGA, a biodegradable and safe synthetic polymer, was used to encapsulate hydrophobic drug Enz (PLGA/Enz), which can improve the drug loading efficiency. Next, CT/mi26a was incubated with Enz-loaded PLGA nanoparticles, forming the inner core structure. Further, to obtain tumor-specific active targeting function [17], the nanoparticles cores were incorporated into BMSC membranes to form a Bm@PT/Enz-miR26a biomimetic nano-system. Our previous studies have demonstrated that BMSC membranes have significant tumor tropism and inflammatory migration properties, containing various receptors and antigens on their surface that can precisely target cancer cells in bone metastasis sites [19]. The electrophoretic mobility shift assay indicated that miR26a could be completely adsorbed onto nanoparticles when the N/P ratio was 0.5 (Fig. 2A). The overall in vitro gene transfection efficiency evaluated in HEK-293T cells showed that higher transfection efficiencies were obtained at an N/P ratio of 20 compared to that of PEI (Fig. 2B-C). An optimal unimodal size distribution of Bm@PT nanoparticles was obtained with a preferably average particle size of ∼ 200 nm diameter and a negative surface charge of ∼ -20 mV when the molar ratio of PLGA: CT peptides was 50:1 and the weight ratio of PT nanoparticles: BMSC membranes was 4:1 (Fig. 2D-E). Furthermore, when visualized with TEM, Bm@PT showed a core-shell structure with an outer shell ∼ 25 nm in thickness, indicating that the BMSC membrane was successfully translocated to the surface of the nanoparticles (Fig. 2F). The stability of the entire biomimetic nano-system was extended in both 1× PBS and 0.5× FBS (Supporting Fig. S5A). And the viability of C4-2B EnzR, C4-2B, and RWPE-1 cell lines was above 80% when incubated with Bm@PT for 48 h (Supporting Fig. S5B). Notably, the diameter and zeta potential of Bm@PT/Enz-miR26a were comparable to those of blank Bm@PT nanoparticles (Supporting Fig. S6A-D), indicating that no significant change in particle size and morphology occurred after drug loading.

Then, to verify the integrity of the biomimetic nano-system, polymeric cores and BMSC membranes were dyed with DiD and DiO, respectively. The resulting dual-fluorophore-labeled nanoparticles were internalized by C4-2B EnzR cells, which displayed a stable core-shell structure (Supporting Fig. S5C). The nanoparticles obtained a high encapsulation efficiency (%) of ∼ 80%, while the drug loading (%) was ∼ 16%. The cytotoxicity assay revealed no apparent cytotoxic effect on the C4-2B EnzR, C4-2B, and RWPE-1 cells.

### BMSC-coated nanoparticles enhanced cellular uptake and intracellular distribution in Enz-resistant cells and promoted lysosomal escape

Next, we investigated the cellular uptake of Bm@PT-based nanoparticles into C4-2B EnzR cells. Nile and miFAM were selected as model drugs that were loaded into Bm@PT [19]. The High-Content Analysis results showed that red and green fluorescence were mainly distributed around the nucleus (DAPI blue staining) and merged into orange fluorescence in the cytoplasm in all groups. Bm@PT/Nile-miFAM group showed significant orange fluorescence compared to the uncoated PT/Nile-miFAM group (Fig. 2G). Moreover, the quantitative measurements by flow cytometry also demonstrate the significantly high cellular uptake efficiency of Bm@PT/Nile-miFAM. The mean fluorescence intensities of Bm@PT/Nile (Fig. 2H) and Bm@PT/miFAM (Fig. 2I) were 1.3 and 3 times higher than those of the uncoated groups, respectively. To further explore the intracellular transport of BMSC-coated nanoparticles, LysoTracker Red was used to label lysosomes in resistant cells. The images obtained from the High-Content Analysis System showed that the green fluorescence of coumarin-6 (C6) overlapped with the red fluorescence of lysosomes around the blue fluorescence of the nucleus after 1 h of incubation. After 4 h, a separation of green and red fluorescence was observed, indicating the high lysosomal escape capacity of the biomimetic nano-system (Fig. 2J). Therefore, these findings demonstrated that BMSC membranes coated on the surface of nanoparticles could cross the lysosomal barrier and deliver the encapsulated agents into the cytoplasm, thereby enhancing the efficiency of targeted drug delivery.

### MiR26a resensitized Enz-resistant cells and synergistically suppressed cell growth in vitro

**Fig. 3:**
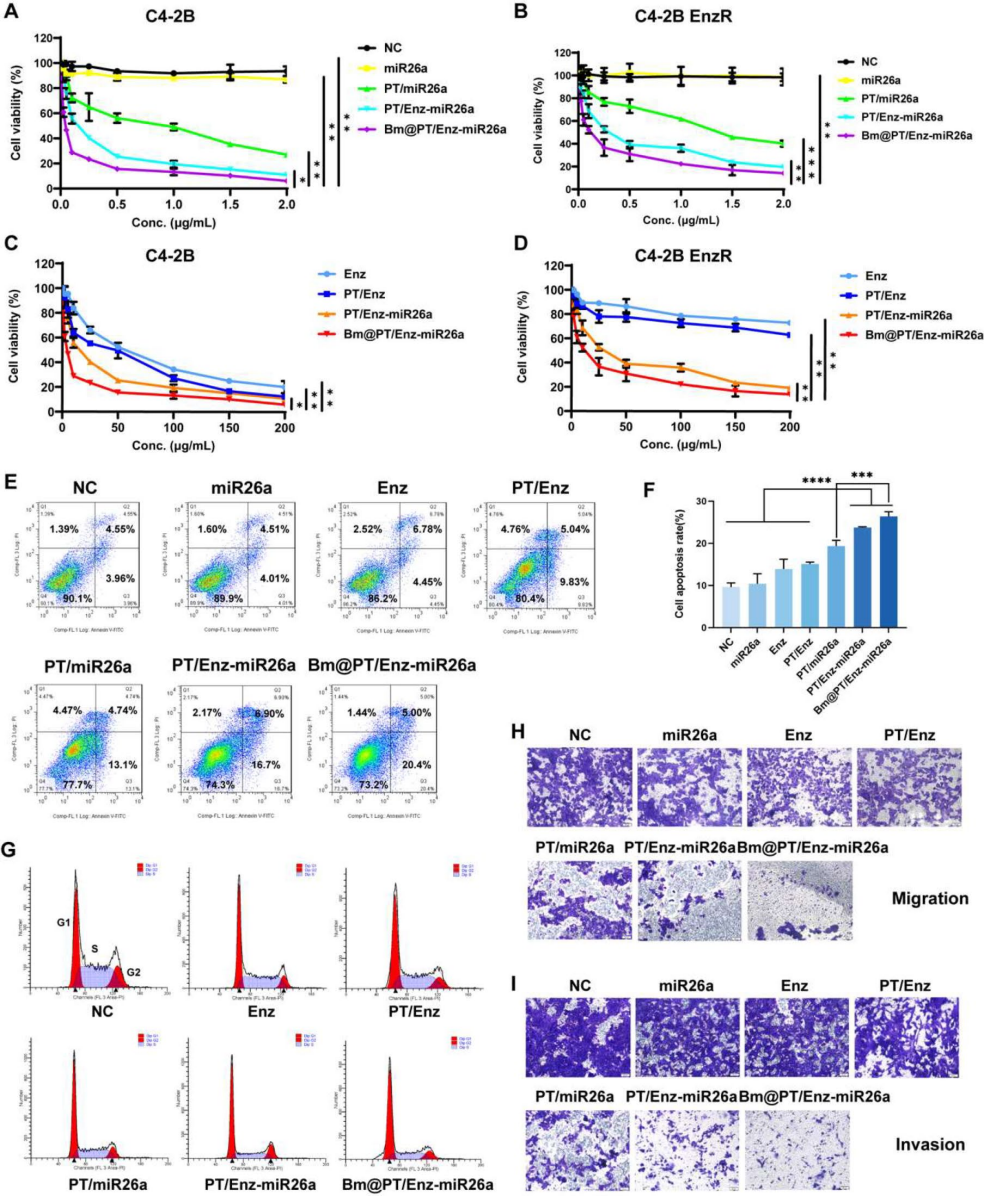
In vitro efficacy of Bm@PT/Enz-miR26a. **(A–D)** MiR26a and Enz synergistically inhibited CRPC cell proliferation. Significant decrease in cell viability when C4-2B and C4-2B EnzR cells were treated with Bm@PT/Enz-miR26a for 48 h compared with other groups (Enz: 0 ∼ 200 µg·mL^− 1^, miR26a: 0 ∼ 2 µg·mL^− 1^). *n* = 3, mean ± SD. **(E)** The representative results of apoptosis of C4-2B EnzR cells treated with each group for 48 h. (Enz: 30 µg·mL^− 1^, miR26a: 0.25 µg·mL^− 1^). **(F)** Statistical results of cell apoptosis in each group. *n* = 3, mean ± SD, ***p < 0.001, ****p < 0.0001, one-way ANOVA. **(G)** Combination therapy with miR26a and Enz induced G0/G1 cell cycle arrest (Enz: 25 µg·mL^− 1^, miR26a: 0.2 µg·mL^− 1^). **(H–I)** Microscope images of anti-migration effects and anti-invasion effects in each group (visualized with 0.1% crystal violet, scale bar: 50 μm). C4-2B EnzR cells were co-incubated with each group for 24 h (anti-migration assay) and 48 h (anti-invasion assay), respectively (Enz: 50 µg·mL^− 1^, miR26a: 0.5 µg·mL^− 1^)

To evaluate the intracellular effects of miR26a on Enz-resistant CRPC, C4-2B EnzR cells, and C4-2B cells were treated with different formulations of the drug to investigate the drug cytotoxicity in vitro. Free miR26a and its negative control (NC) showed no apparent toxicity to either cell line (Fig. 3A-B). In addition, the free Enz (IC_50_ = 52.65 µg·mL^− 1^) and PT/Enz (IC_50_ = 30.59 µg·mL^− 1^) groups exhibited dose-dependent cytotoxicity against C4-2B cells but showed slight cytotoxicity to C4-2B EnzR cells (Fig. 3C-D). These results also demonstrated that neither Enz alone nor Enz-loaded nanoparticles could suppress the viability of C4-2B EnzR cells (Resistance index = 24.1). As expected, the PT/miR26a, PT/Enz-miR26a, and Bm@PT/Enz-miR26a groups showed significant cytotoxicity against both cell lines with increasing concentrations (Fig. 3A-B). In particular, the Bm@PT/Enz-miR26a group (Enz/miR26a: IC_50_ = 11.83/0.12 µg·mL^− 1^) (Supporting Table S1) exhibited the highest cytotoxicity, which was 3 times higher than that of the PT/Enz-miR26a group. A similar tendency of cytotoxicity was obtained against C4-2B cells. MiR26a synergistically enhanced the toxicity of Enz in the two cell lines, and the increase in cytotoxicity may be attributed to the function of miR26a in reversing Enz resistance as well as the improved cellular uptake of BMSC-coated nanoparticles. Apoptosis assays further confirmed the anti-proliferation ability of Bm@PT/Enz-miR26a. The cell apoptosis rate determined by flow cytometry showed that the average apoptosis rate of C4-2B EnzR cells treated with Bm@PT/Enz-miR26a was the highest and reached 26.4%, which was 1–3 times greater than that of PT/Enz-miR26a and the other groups (Fig. 3E-F).

### Combination therapy with miR26a and Enz induced cell cycle arrest and inhibited cell invasion and metastasis

Multiple studies have demonstrated that both miR26a and Enz could induce G0/G1 cell cycle arrest to block the G1/S phase transition [28–31]. We hypothesized that the combination of miR26a and Enz could synergistically increase the percentage of resistant cells in G0/G1 phase. C4-2B EnzR cells were incubated with miR26a/Enz-loaded nanoparticles, and the cell cycle was analyzed by flow cytometry. The results indicated that simultaneous treatment with miR26a and Enz contributed to a significantly increased percentage of C4-2B EnzR cells in G0/G1 phase and decreased the proportion in S phase compared to that of NC alone and miR26a/Enz (Fig. 3G). Notably, approximately 60% of resistant cells treated with Bm@PT/Enz-miR26a were arrested in G0/G1 phase, which was the highest proportion among the groups (Supporting Fig. S7A). These data illustrated that miR26a and Enz could synergistically inhibit the proliferation of Enz-resistant cells by inducing cell cycle arrest in the G0/G1 phase.

Next, the anti-invasion and anti-metastasis effects were determined by Transwell chamber assays. The images acquired by microscopy showed that the combined groups, including PT/miR26a, PT/Enz-miR26a, and Bm@PT/Enz-miR26a, exhibited higher anti-migration (Fig. 3H) and anti-invasion (Fig. 3I) abilities than those of the other groups. In particular, compared with PT/Enz-miR26a, Bm@PT/Enz-miR26a significantly inhibited the metastasis (****p < 0.0001) (Supporting Fig. S7B) and invasion (****p < 0.0001) (Supporting Fig. S7C) and of C4-2B EnzR cells.

### BMSC-coated nanoparticles improve penetration and antitumor efficacy in a 3D tumor model

**Fig. 4:**
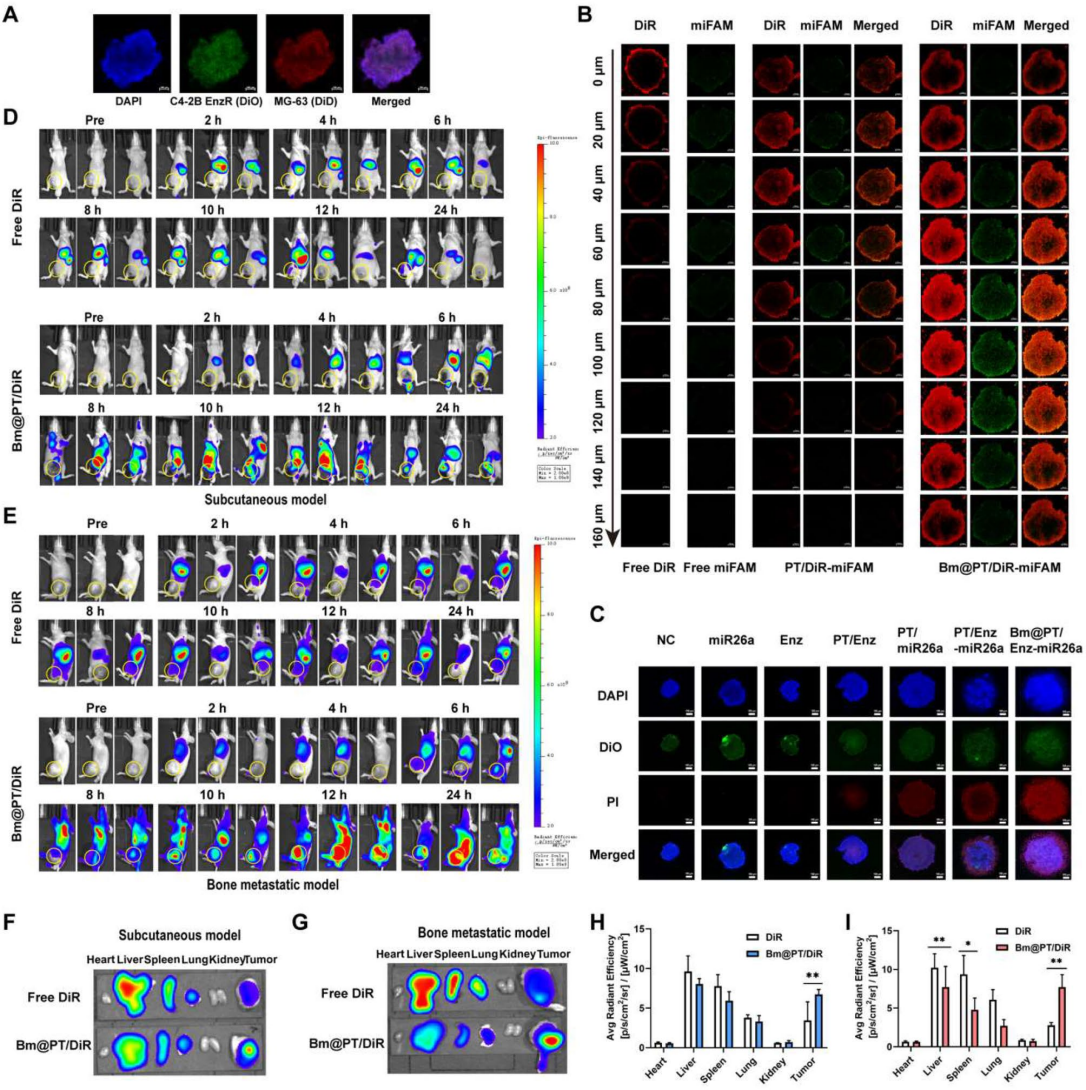
The penetration and antitumor efficacy in 3D tumor models and in vivo biodistribution. **(A)** 3D tumor spheroid model. Blue fluorescence: DAPI, green fluorescence: DiO, red fluorescence: DiD, Scale bar: 100 μm. **(B)** The images of in vitro tumor spheroids penetration of each group were acquired by CLSM at 10× objective magnification using Z-stack imaging from the bottom of the spheroids at 20 μm intervals. DiO-stained C4-2B EnzR cells and DiD-stained MG-63 cells were seeded in the 96-well plates at the ratio of 1:1. (DiR: 20 µg·mL^− 1^, miFAM: 0.5 µg·mL^− 1^). Scale bar: 100 μm. **(C)** The tumor spheroids with a diameter of about 400 μm were co-incubated with different formulations for 48 h (Enz: 50 µg·mL^− 1^, miR26a: 1 µg·mL^− 1^) and images were acquired by CLSM. Scale bar: 100 μm. Representative small animal living images of the biodistribution of each group in **(D)** subcutaneous CRPC and **(E)** BmCRPC mouse models at 0–24 h. Free DiR and DiR-loaded Bm/PT nanoparticles were injected at a concentration of 10 mg·kg^− 1^. **(F)** Distribution in major organs of each group in subcutaneous CRPC and **(G)** BmCRPC mouse models. Quantified distribution in major organs of each group in **(H)** subcutaneous CRPC and **I.** BmCRPC mouse models. *n* = 3, mean ± SD, *p < 0.05, **p < 0.01, one-way ANOVA

To better simulate the in vivo bone metastatic microenvironment, we established a 3D tumor spheroid model containing C4-2B EnzR cells and MG-63 osteosarcoma cells (Fig. 4A). To determine whether the BMSC-coated nano-system could enhance drug penetration in spheroids, DiR and miFAM were loaded into PT and Bm@PT to mimic Enz and miR26a. The 3D tumor spheroids were treated with different formulas, and the penetration process was photographed by CLSM. Both DiR and miFAM were mainly distributed in the periphery of the tumor spheroids. Notably, DiR and miFAM in the Bm@PT/DiR-miFAM group deeply penetrated into the core of the tumor spheroids, and the fluorescence intensity was obviously higher than that in the PT/DiR-miFAM group when the depths reached 160 μm (Fig. 4B), indicating that Bm@PT exhibited a high capability of tumor penetration.

The tumor spheroids were then exposed to different formulations, and the antitumor efficacy was evaluated *via* a Live/Dead kit. As shown by CLSM, the red fluorescence intensity of the PT/miR26a, PT/Enz-miR26a, and Bm@PT/Enz-miR26a groups was higher than that of the control, free Enz, free miFAM, and PT/Enz groups. Obviously, the Bm@PT/Enz-miR26a group showed the highest red fluorescence intensity, indicating its high antitumor efficacy (Fig. 4C).

### BMSC-coated nanoparticles efficiently targeted delivery of Enz and miR26a to tumor sites

Following the findings in 2D and 3D cell models, subcutaneous CRPC and BmCRPC mouse models were established to investigate in vivo biodistribution. DiR was loaded into Bm@PT to trace the in vivo biodistribution. The DiR fluorescence of the Bm@PT/DiR group began to accumulate at the tumor site at 6 h, and the fluorescence intensity continued to increase, reaching a maximum at 12 h in both subcutaneous (Fig. 4D) and bone metastatic models (Fig. 4E). The decrease in fluorescence at 24 h may mainly be due to hepatic metabolism. Additionally, it was shown that low fluorescence appeared at tumor sites in the free DiR group within 0–24 h, with a large amount of DiR accumulating in the liver. After 24 h of observation, the mice were sacrificed to quantify the biodistribution of fluorescence in different organs. The Bm@PT/DiR group showed obvious fluorescence intensities in both subcutaneous (Fig. 4F) and bone metastatic tumor sites (Fig. 4G), while the fluorescence intensity at the tumor site in the free DiR group was much lower. Moreover, the accumulation of fluorescent nanoparticles in the liver, spleen, and lung was reduced in the Bm@PT/DiR group compared with the free DiR group in subcutaneous (**p < 0.01) (Fig. 4H) and bone metastatic tumors (**p < 0.01) (Fig. 4I). These data confirmed that BMSC-coated carriers showed a better ability to deliver drugs to tumor sites, prolong circulation and reduce accumulation in the liver and spleen.

### MiR26a reversed Enz resistance and synergistically inhibited tumor growth and distant organ metastasis in vivo

**Fig. 5:**
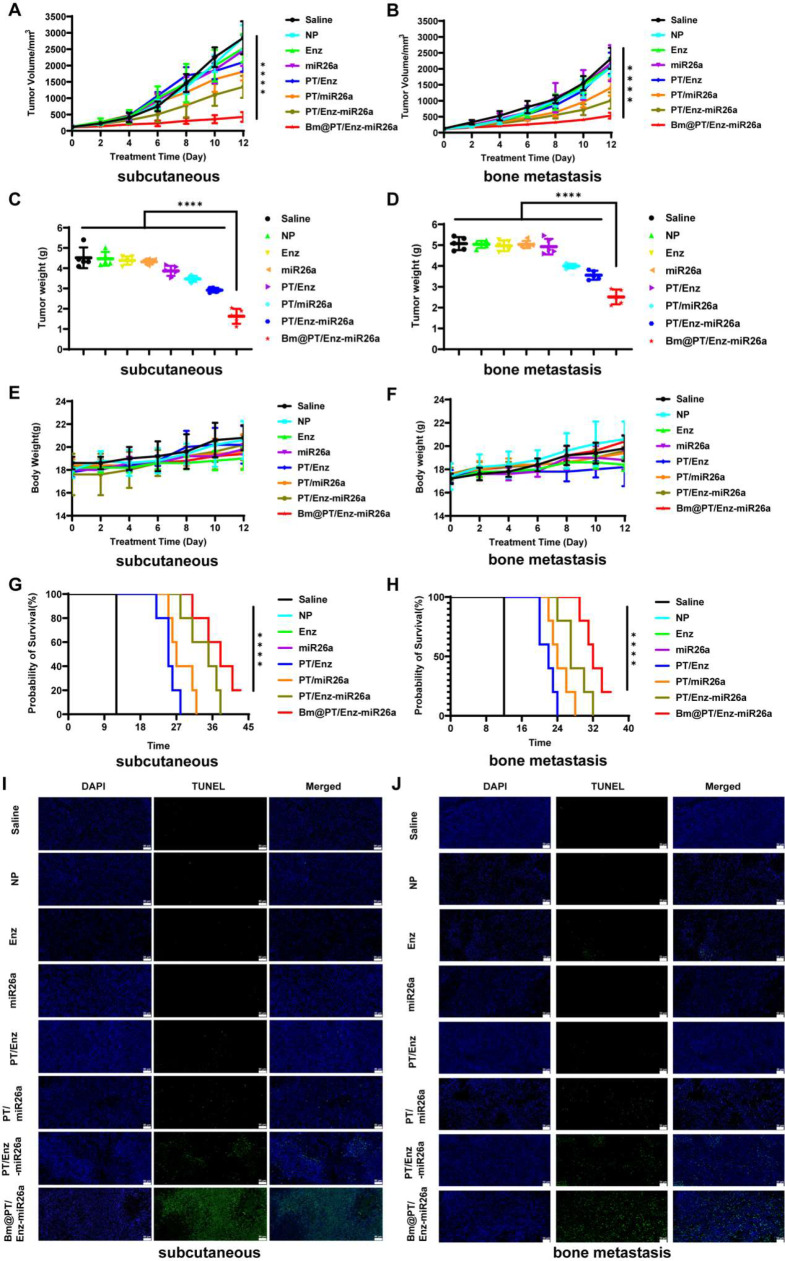
In vivo therapeutic effects of Bm@PT/Enz-miR26a in two models. **(A–B)** Tumor volume growth in subcutaneous CRPC and BmCRPC mouse models over 12 days. *n* = 5, mean ± SD, **p < 0.01, ***p < 0.001, ****p < 0.0001, one-way ANOVA. **(C–D)** Tumor weight growth in subcutaneous CRPC and BmCRPC mouse models. *n* = 5, mean ± SD, ****p < 0.0001, one-way ANOVA. **(E–F)** Body weight growth in subcutaneous CRPC and BmCRPC mouse models over 12 days. *n* = 5, mean ± SD. **(G–H)** Survival time of tumor-bearing mice in subcutaneous CRPC and BmCRPC mouse models. *n* = 5, mean ± SD, ****p < 0.0001, one-way ANOVA. **(I–J)** TUNEL assay was performed to evaluate the apoptosis of tumor cells in subcutaneous CRPC and BmCRPC mouse models. TUNEL: green, Scale bar: 50 μm

Based on the preferential targeted accumulation in tumors, we further investigated the in vivo therapeutic effects of combination therapy using these two models. Because the tumor size of the mice in the saline, NP, and Enz group exceeded ethical principles and were sacrificed on day 12, we only counted the tumor volume and weight of the mice over 12 days. Similar to the saline group, blank nanoparticles (NP), free Enz, and free miR26a failed to retard tumor growth in both models. Inhibition of tumor growth was noted in the PT/miR26a, PT/Enz-miR26a, and Bm@PT/Enz-miR26a groups. Notably, tumor-bearing mice treated with Bm@PT/Enz-miR26a showed the strongest antitumor effect (Fig. 5A-B). The tumor weight of mice treated with Bm@PT/Enz-miR26a was ∼ 1.8-3-fold and ∼ 1.4-2-fold lighter than those treated with the other groups in the two models, respectively (Fig. 5C-D). During the treatment period, the mice in these two models exhibited a similar steady increase in body weight (Fig. 5E-F). What’s more, tumor-bearing mice treated with Bm@PT/Enz-miR26a had longer median survival time which was 38 days in subcutaneous CRPC mouse model (Fig. 5G) and 32 days in BmCRPC mouse model (Fig. 5H). Moreover, a TUNEL assay was performed to evaluate the apoptosis of tumor cells. Compared with the PT/miR26a and PT/Enz-miR26a groups, more green fluorescent cells were observed in the Bm@PT/Enz-miR26a group, suggesting that apoptosis was significantly induced. Additionally, no apparent TUNEL-positive cells were observed in other groups (Fig. 5I-J).

**Fig. 6:**
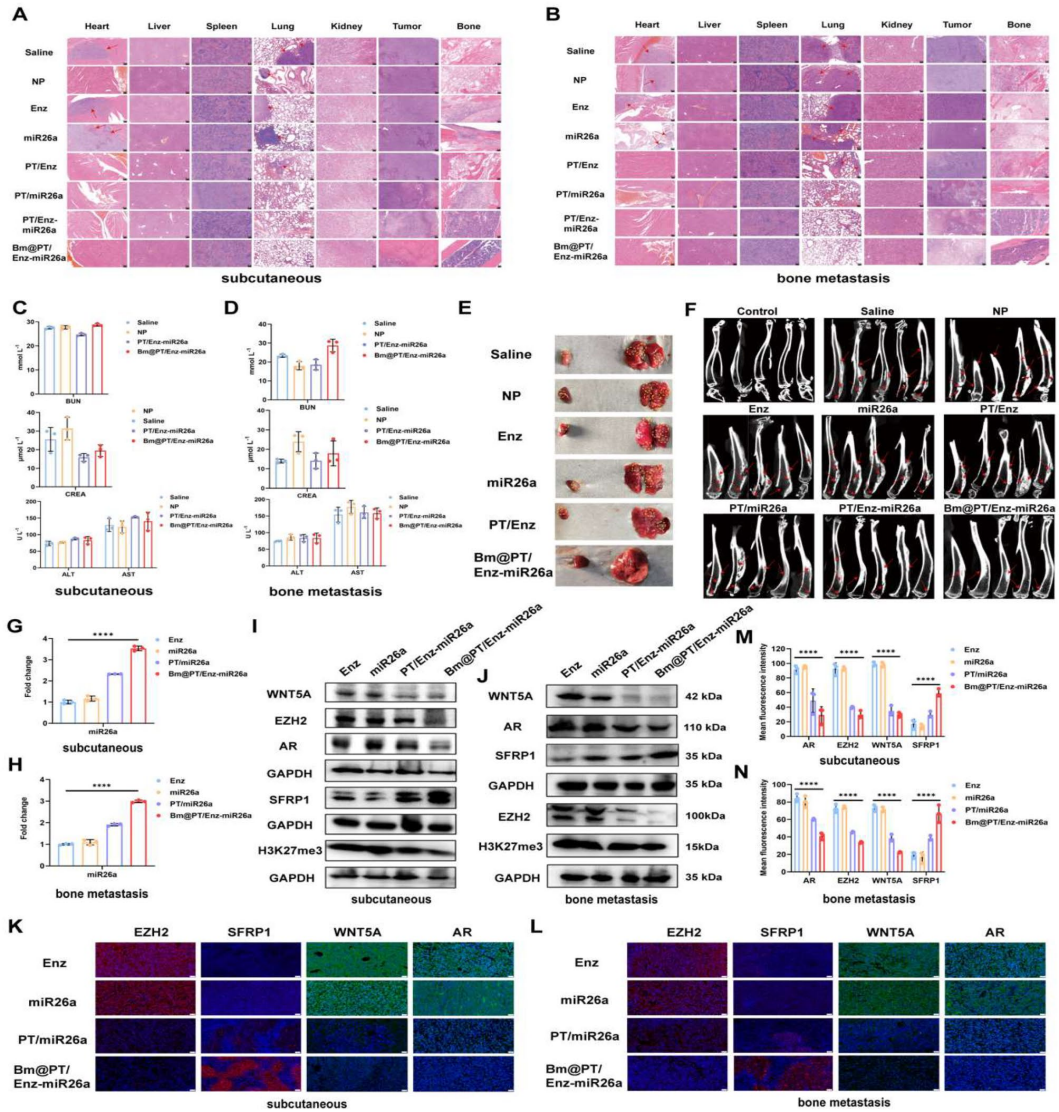
In vivo safety and bone protection of Bm@PT/Enz-miR26a in two models. **(A–B)** The H&E images of heart, liver, spleen, lung, kidney, tumor, and bone (left hind limb tibia) of each group in **(A)** subcutaneous CRPC and **(B)** BmCRPC mouse models. Scale bar: 100 μm. Red arrow: tumor metastatic sites. **(C–D).** The biochemical analysis of mouse blood samples in **(C)** subcutaneous CRPC and **(D)** BmCRPC mouse models. *n* = 3, mean ± SD. **(E)** Metastasis in the heart and lungs of tumor-bearing mice in BmCRPC model. Yellow circle: tumor. **(F)** The micro-CT images of each group in the BmCRPC mouse model. *n* = 5, red arrows: bone damaged sites. **(G–H)** The miR26a level of each group in **(G)** subcutaneous CRPC and **(H)** BmCRPC mouse models. *n* = 3, mean ± SD, ****p < 0.0001, one-way ANOVA. **(I–J)** Related protein expressions of each group in **(I)** subcutaneous CRPC and **(J)** BmCRPC mouse models. **(K)** Immunofluorescence results of each group in subcutaneous CRPC model. Scale bar: 20 μm. **(L)** Immunofluorescence results of each group in the BmCRPC mouse model. Scale bar: 20 μm. **(M)** Mean fluorescence intensity in subcutaneous CRPC model. *n* = 3, mean ± SD, ****p < 0.0001, one-way ANOVA. **(N)** Mean fluorescence intensity in BmCRPC mouse model. *n* = 3, mean ± SD, ****p < 0.0001, one-way ANOVA

Furthermore, in all groups, H&E staining of major organs, including heart, liver, spleen, lung, and kidney, exhibited no obvious histological toxicity, indicating that this biomimetic nano-system exhibited good biocompatibility and tissue tolerance (Fig. 6A-B). Consistent with the above results, the biochemical analysis of mouse blood samples revealed that serum aspartate aminotransferase (AST), alanine aminotransferase (ALT), blood urea nitrogen (BUN), and creatinine (CR) levels in saline, NP, PT/Enz-miR26a, and Bm@PT/Enz-miR26a groups were all within the normal range in these two models (Fig. 6C-D).

More importantly, tumor metastatic lesions were found in the hearts and lungs of both models in saline, NP, miR26a, Enz, and PT/Enz groups, and tumor metastatic lesions in the BmCRPC mouse model were larger and more numerous than those in the subcutaneous CRPC mouse model. No secondary organ metastasis was apparent in PT/miR26a, PT/Enz-miR26a, and Bm@PT/Enz-miR26a groups (Fig. 6E). Moreover, histology showed that tumor cells in Bm@PT/Enz-miR26a group were significantly reduced, and no apparent nuclei were observed compared to that of the other groups. In addition, H&E staining of mouse tumor-bearing tibia showed severe bone damage and loss, enlarged intercellular spaces, increased cytoplasm, and blurred boundaries between bone and bone marrow in saline, NP, free Enz, free miR26a, and PT/Enz groups. However, PT/miR26a and PT/Enz-miR26a groups incurred less bone damage and loss. It is worth noting that in the Bm@PT/Enz-miR26a group, there was no apparent bone damage in the tumor-bearing tibias (Fig. 6A-B). Subsequently, microCT was applied to assess bone loss. The skeletal structure of mice treated with Bm@PT/Enz-miR26a maintained an intact cortical shell, while the other groups exhibited deformations and even fractures due to osteolysis (Fig. 6F). The BMD value of Bm@PT/Enz-miR26a group was slightly lower than that of the control group, but the difference was not significant (*p* > 0.05). In contrast, the BMD values of the other groups were significantly lower than those of the control group (*p < 0.05), indicating severe bone loss (Supporting Fig. S8A-B). These results demonstrated that Bm@PT/Enz-miR26a not only inhibited the growth and metastasis of Enz-resistant CRPC tumors but also effectively inhibited the secondary organ metastasis of BmCRPC.

### MiR26a in combination with Enz exerted antitumor growth and antimetastatic effects via the EZH2/SFRP1/WNT5A axis in vivo

To further identify the mechanism of miR26a combined with Enz in vivo, PCR and western blotting were used to detect the expression level of miR26a and related genes in tumor tissues. MiR26a expression was significantly increased in the Bm@PT/Enz-miR26a group (****p < 0.0001), which was 1.5-3.5-fold higher than that in the Enz, miR26a, and PT/miR26a groups in subcutaneous tumor tissues (Fig. 6G). A similar result was observed in the BmCRPC mouse model. The expression of miR26a in the Bm@PT/Enz-miR26a group was 1.5-3-fold higher than in other groups (Fig. 6H). Western blotting results showed that the expression levels of EZH2, H3K27me3, WNT5A, and AR were markedly decreased, while SFRP1 was upregulated in the Bm@PT/Enz-miR26a group compared with other groups in these two models (Fig. 6I-J; Supporting Fig. S8C-D). Moreover, immunofluorescence assays demonstrated that these genes were mainly localized in the cytoplasm. Tumor-bearing mice treated with Bm@PT/Enz-miR26a showed significant fluorescence of SFRP1 and decreased fluorescence of EZH2, WNT5A, and AR compared to other groups. Further statistical analysis of the mean fluorescence intensity of each group confirmed these results (Fig. 6K and M). The results of the immunofluorescence assay were consistent with those of the BmCRPC model (Fig. 6L and N).

The above results illustrated that Bm@PT/Enz-miR26a could significantly increase the expression of miR26a in Enz-resistant CRPC cells and tumor tissues and further inhibit tumor growth and metastasis *via* EZH2/SFRP1/WNT5A axis, which restored the sensitivity of Enz-resistant tumor cells to Enz and inhibited the transcriptional activation and expression of AR.

## Discussion

The AR inhibitor Enz is the first-line treatment for CRPC. However, CRPC patients often develop resistance to Enz through multiple mechanisms, including AR reactivation, AR mutation, and epigenetic reprogramming [31, 32]. Moreover, about 80–90% of CRPC patients develop bone metastasis, since the PCa cells expressing the E-selectin ligand CD44, αvβ3 integrin, and CXCR4 bind to bone marrow endothelial cells, bone marrow stromal cells and mesenchymal stem cells to promote bone homing of PCa [33, 34]. BmCRPC is generally incurable with limited options for treatment as well as decreased quality of life, including bone pain and bone destruction [35].

WNT5A and EZH2 play crucial roles in the development of Enz resistance and the metastasis of CRPC. After Enz treatment, WNT5A is induced to increase CTCs in CRPC patients, and its ectopic expression attenuates the effect of AR inhibitors [13]. EZH2 acts as a transcriptional activator in CRPC to directly induce *AR* gene expression in a polycomb-and methylation-independent manner [8]. EZH2 also regulates tumor heterogeneity, promotes Enz resistance, and mediates epigenetic reprogramming to promote secondary metastasis from bones to other organs [9]. Therefore, down-regulating WNT5A or EZH2 could inhibit Enz resistance and BmCRPC. Based on the TargetScan database, we identified miR26a could block WNT5A or EZH2 directly (Fig. 1D). Further, the in vitro and in vivo tests also demonstrated that miR26a targets the 3’UTR of EZH2 and WNT5A mRNAs to down-regulate their expressions (Fig. 6I, J).

Moreover, our bioinformatic analysis showed that the WNT5A antagonist SFRP1 was decreased in metastatic PCa patients treated with Enz. EZH2 was reported to mediate *PGC1α* gene silencing, thereby increasing WNT5A expression and promoting migration and metastasis in melanoma [36]. To investigate whether there is an interaction between EZH2 and WNT5A in prostate cancer, we transfected C4-2B EnzR cells with siEZH2, siSFRP1, and their negative controls. The results indicated that EZH2 directly downregulated SFRP1 expression, and WNT5A was directly regulated by SFRP1. The rescue experiment further demonstrated the regulatory effects of the EZH2/SFRP1/WNT5A axis (Fig. 1E-H). Thus, we speculated that miR26a may retrieve the sensitivity of Enz to CRPC and inhibit BmCRPC by modulating EZH2/SFRP1/WNT5A axis. Notably, WNT5A expression in the siNC1-treated group was not statistically different from that in the siEZH2-treated group. Previous studies demonstrated that EZH2 could silence the expression of WNT5A in colon cancer [37]. This may be the reason for the upregulation of WNT5A and needs to be further verified in BmCRPC. To our knowledge, this is the first study to investigate the EZH2/SFRP1/WNT5A axis in Enz-resistant CRPC.

To target the delivery of Enz and miR26a to cancer and bone metastasis, we designed Bm@PT/Enz-miR26a. The in vivo/in vitro results demonstrated that Bm@PT/Enz-miR26a provides distinct advantages, including high drug loading, high gene transfection, targeted abilities, prevention of intracellular lysosomal degradation, and clearance with low toxicity and enhanced stability. Moreover, Bm@PT/Enz-miR26a increased apoptosis in Enz-resistant CRPC cells, significantly inhibited cell invasion and metastasis, and blocked cells in G0/G1 phase in 2D cell models with enhanced tumor penetration and drug delivery to the deep layers of 3D tumor spheroids (Fig. 4A-C). Further, the combination of Enz and miR26a in Bm@PT showed a synergistic effect in inhibiting the growth of Enz-resistant CRPC and bone metastasis. In subcutaneous CRPC and BmCRPC mouse models, Bm@PT/Enz-miR26a exhibited the highest tumor regression synergy in all groups, with good biosafety. In addition, mice treated with Bm@PT/Enz-miR26a maintained a skeletal structure with an intact cortical shell and the median survival time was prolonged in the two mouse models (Fig. 5).

Interestingly, we found that inhibiting EZH2 could suppress secondary metastasis from bone to other organs. Similar results were reported by Lege et al. that enhanced EZH2 activity mediated increased stemness and metastasis capacity, promoting further breast cell metastasis and establishing multi-organ secondary metastasis [9, 10]. In our study, H&E-stained anatomical photographs of tissues and organs in the BmCRPC mouse model clearly showed that the heart and lung metastasis in the control group were larger and more numerous than those in the subcutaneous CRPC mouse models (Fig. 6A). No secondary organ metastasis was apparent in the groups including miR26a (PT/miR26a, PT/Enz-miR26a, and Bm@PT/Enz-miR26a) in the BmCRPC mouse model (Fig. 6B). These results indicated that miR26a could block the secondary metastasis from bone to other organs such as the heart and lung.

## Conclusions

In summary, we identified that miR26a could target WNT5A and EZH2 directly. Further, we constructed Bm@PT nanoparticles for the co-delivery of Enz and miR26a. The in vivo/in vitro results demonstrated that Bm@PT/Enz-miR26a provides distinct advantages, including high drug loading, high gene transfection, targeted abilities, prevention of intracellular lysosomal degradation, strong efficacy, and good biocompatibility. Moreover, the upregulation of miR26a could restore Enz sensitivity *in vitro and in vivo*, synergistically suppressing tumor growth, bone metastasis, and secondary metastasis by regulating the EZH2/SFRP1/WNT5A axis. Therefore, this research provides a potential new combination therapy strategy for Enz-resistant CRPC and BmCRPC.

### Electronic supplementary material

Below is the link to the electronic supplementary material.

Supplementary Material 1

## Data Availability

The authors declare that all data supporting the results of this study are available within the paper and its supplemental information files. Source data collected in this study are available from the corresponding author upon request.
