# Supplementary material for: MiR26a reverses enzalutamide resistance in a bone-tumor targeted system with an enhanced effect on bone metastatic CRPC
Source: J Nanobiotechnology. 2024 Apr 2;22:145. doi: 10.1186/s12951-024-02438-z (PMC10985917; doi:10.1186/s12951-024-02438-z)
Supplement: Supplementary file 1 — Supplementary Material 1 [file 12951_2024_2438_MOESM1_ESM.docx]

**MiR26a Reverses Enzalutamide Resistance by Inhibiting the EZH2/SFRP1/WNT5A Pathway in a Bone-Tumor Targeted System with an Enhanced Effect on Bone Metastatic CRPC**

*Yuanyuan Wang^a^ †, Jiyuan Chen^b^†,* *Luyao Gong^a^, Yunxia Wang^a^, Aino Siltari^c^, Yan-Ru Lou^a^, Teemu J. Murtolad, Shen Gao^e^, Yuan Gao^a*^*

^a^School of Pharmacy, Fudan University, Shanghai, 201206, China

^b^Department of Pharmacy, Shanghai Ninth People’s Hospital, Shanghai Jiao Tong University School of Medicine, Shanghai, 200011, China

^c^Faculty of Medicine and Health Technology, Tampere University, 33100 Tampere, Finland

^d^TAYS Cancer Center, Department of Urology, Tampere University Hospital, 33100 Tampere, Finland

^e^Department of Pharmacy, Changhai Hospital, Naval Medical University, Shanghai, 200433, China

† These authors contributed equally to the manuscript

^*^ Corresponding author.

Email: yuan_gao@fudan.edu.cn (Y. Gao)


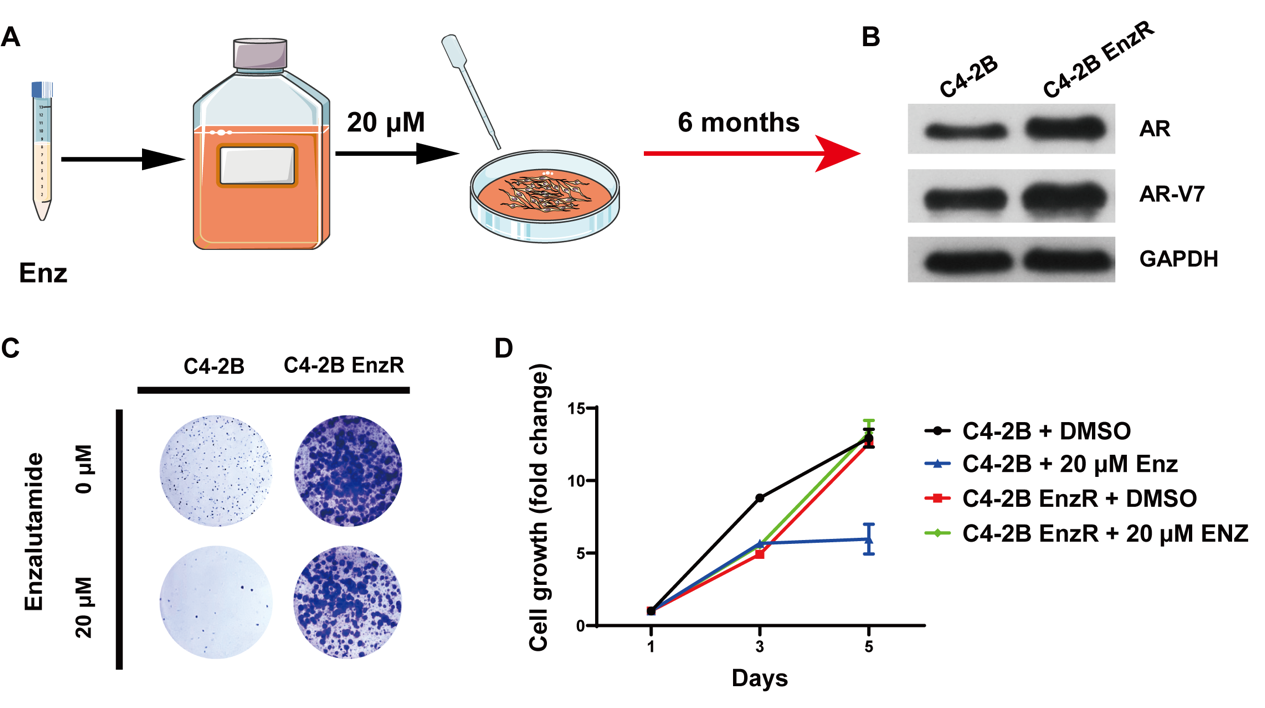


**Fig. S1.** **Establishment and characterization of C4-2B EnzR cells. (A)** Establishment of C4-2B EnzR cells. **(B)** Western blotting results of related proteins in C4-2B EnzR cells compared with C4-2B cells. **(C)** Representative images of colony formation assay (Enz: 20 μM). **(D)** The results of cell growth assay (Enz: 20 μM). n = 3, mean ± SD.

**
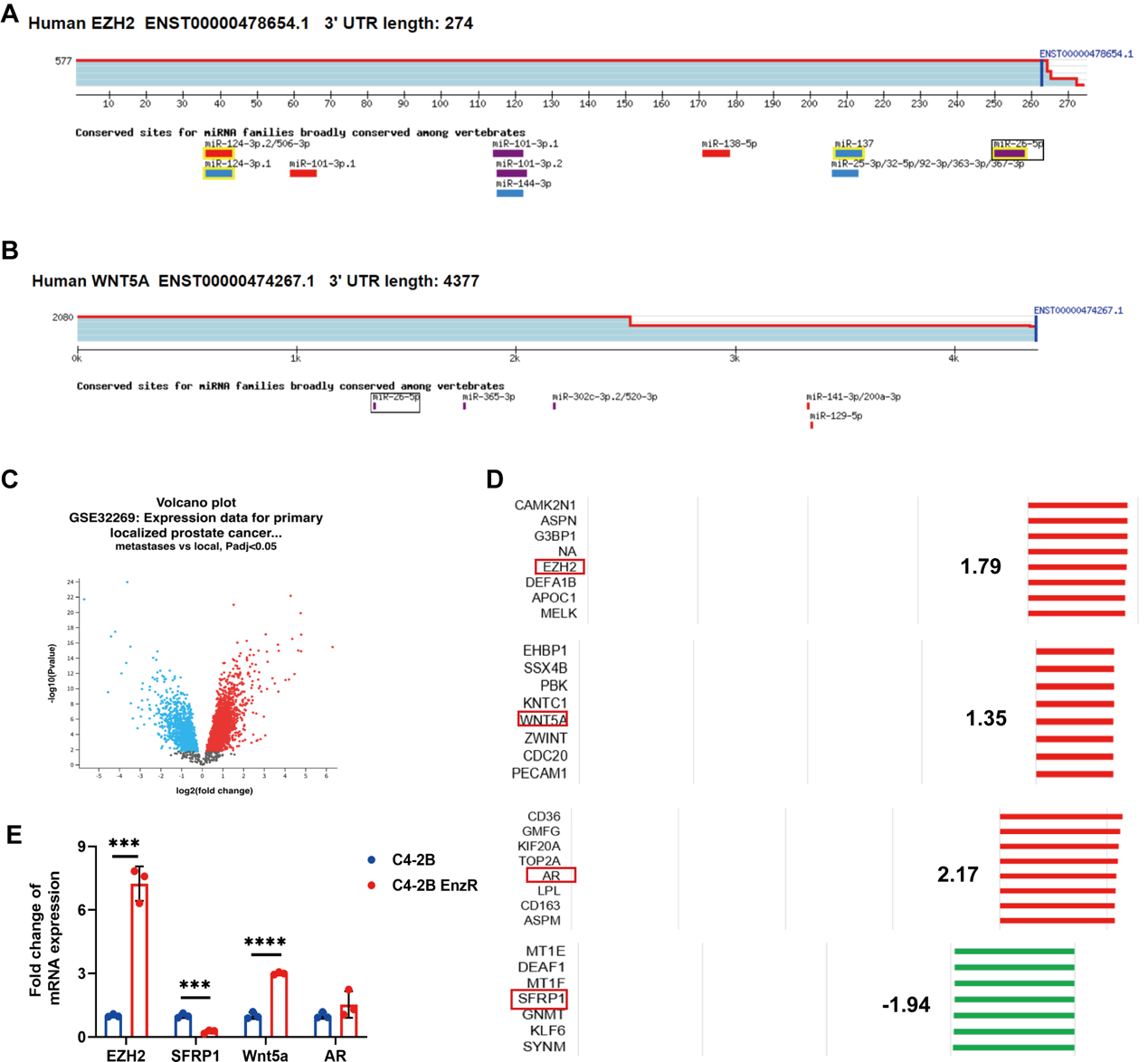
**

**Fig. S2. Bioinformatics analysis of GSE32269 dataset. (A-B)** TargetScan database prediction. miR26a binds to the 3′-UTR of EZH2 and WNT5A mRNA. **(C)** Volcano plot. **(D)** EZH2, WNT5A, and AR were upregulated, while SFRP1 was downregulated after differential expression analysis with the criteria of a false discovery rate (FDR) < 0.05 and more than one-fold change. **(E)** Related gene expressions were detected *via* RT-PCR. n = 3, mean ± SD, ****p* < 0.001, *****p* < 0.0001, one-way ANOVA.


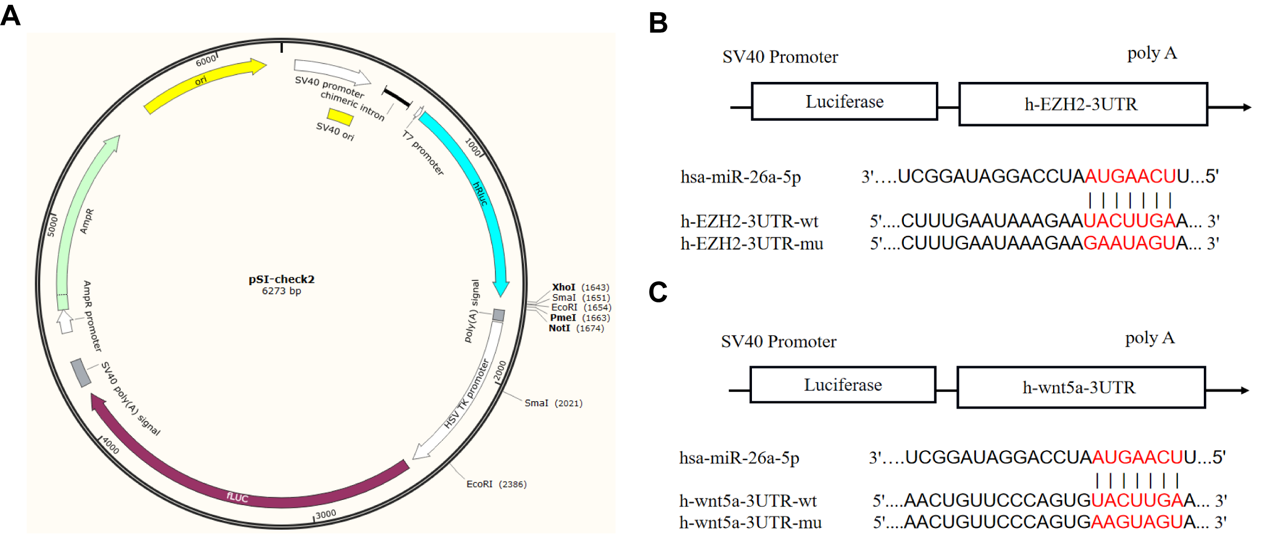


**Fig. S3. Establishment of dual luciferase reporter assays.** (**A).** pSI-Check2 vector map. (**B-C).** Schematic diagram of target combination of hsa-miR-26a-5p, h-EZH2-3UTR, and h-WNT5A-3UTR.

**
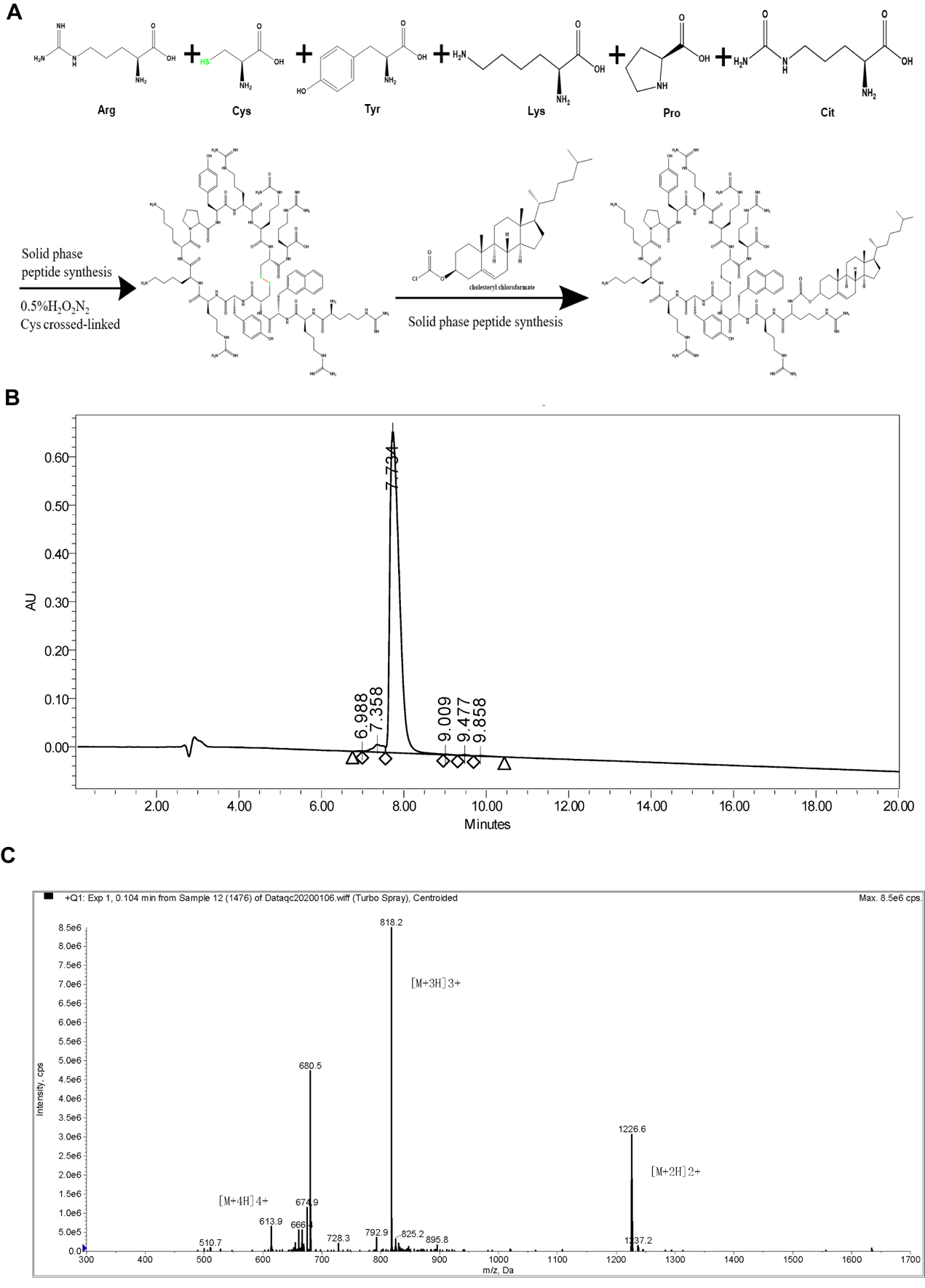
**

**Fig. S4. Synthesis and characterization of CT peptide. (A)** Cholesterol-modified T140 polypeptide was synthesized by solid-phase synthesis. **(B)** The chromatographic peak of CT has a retention time of 7.734 min and **(C)** its molecular weight is 2449.07, which conforms to its structural characteristics. The purity is 96.7%.


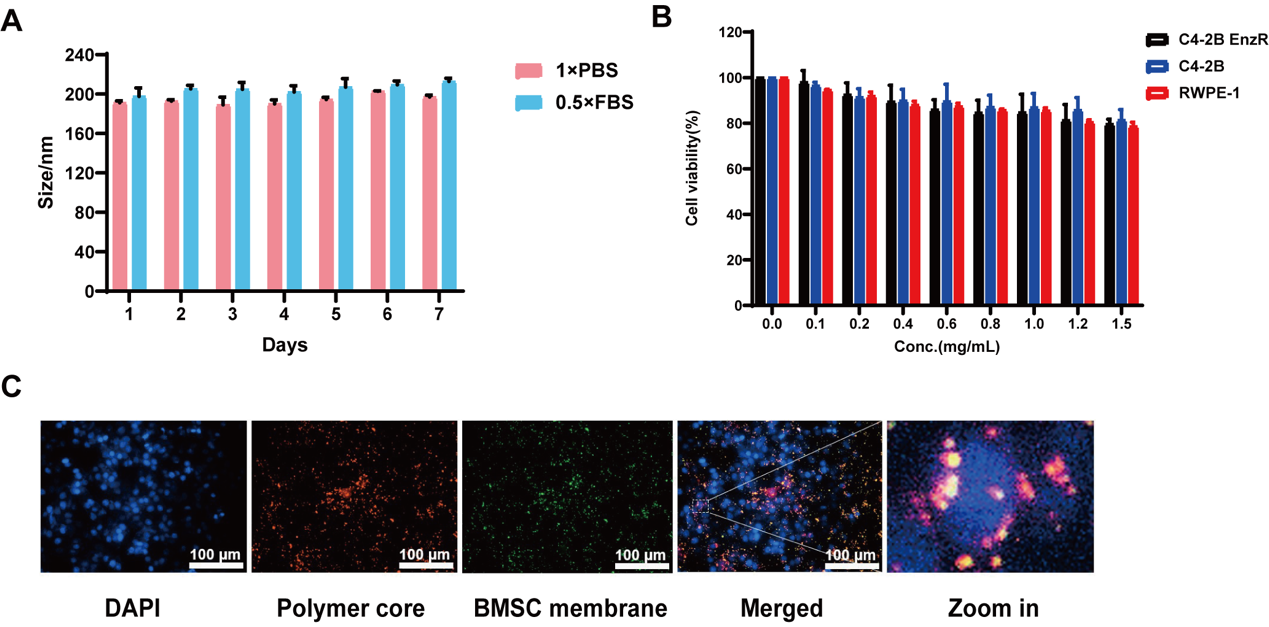


**Fig. S5. characterization of Bm@PT/Enz-miR26a. (A).** The stability of Bm@PT/Enz-miR26a in two solutions. **(B).** Cytotoxicity of Bm@PT nanoparticles on C4-2B, C4-2B EnzR, and RWPE1 cells. n = 3, mean ± SD, Bm@PT: 0~1.5 mg·mL^-1^. **(C).** CLSM images of the colocalization of the BMSC membranes (visualized with green DiO dyes) and polymeric cores (visualized with red DiD dyes) after being internalized by C4-2B EnzR cells. Scale bar: 100 μm.

**
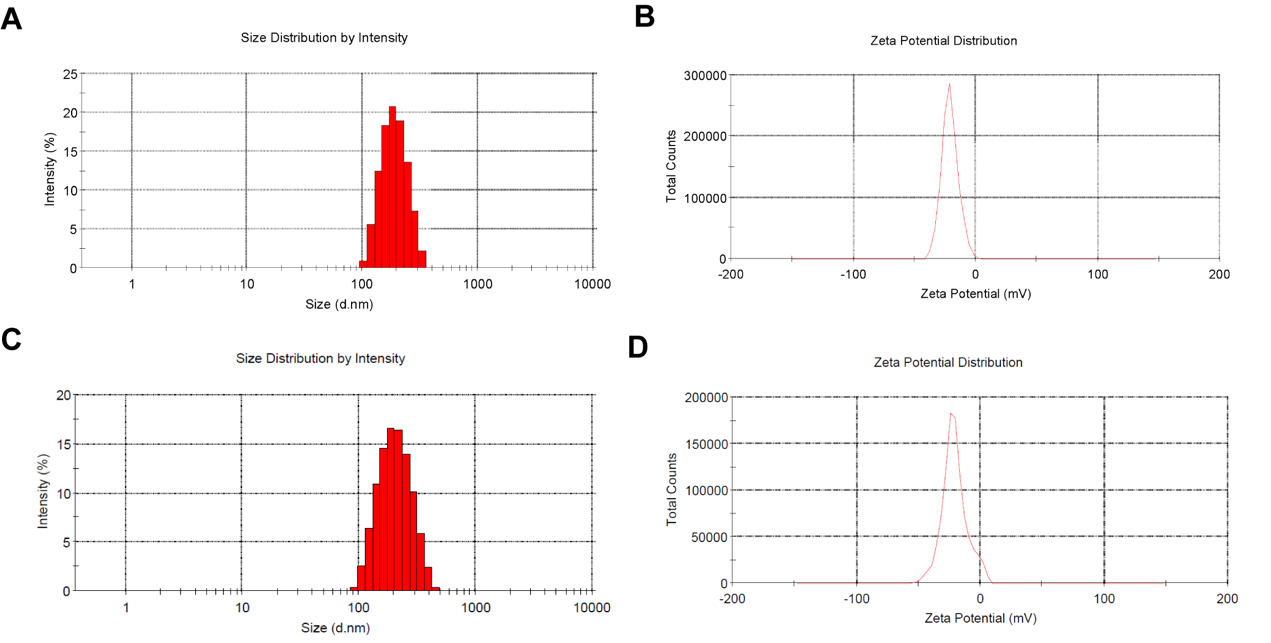
**

**Fig. S6. Characterization of BMSC-coated nanoparticles. (A)** Particle size and **(B)** zeta potential of Bm@PT. **(C)** Particle size and **(D)** zeta potential of Bm@PT/Enz-miR26a.


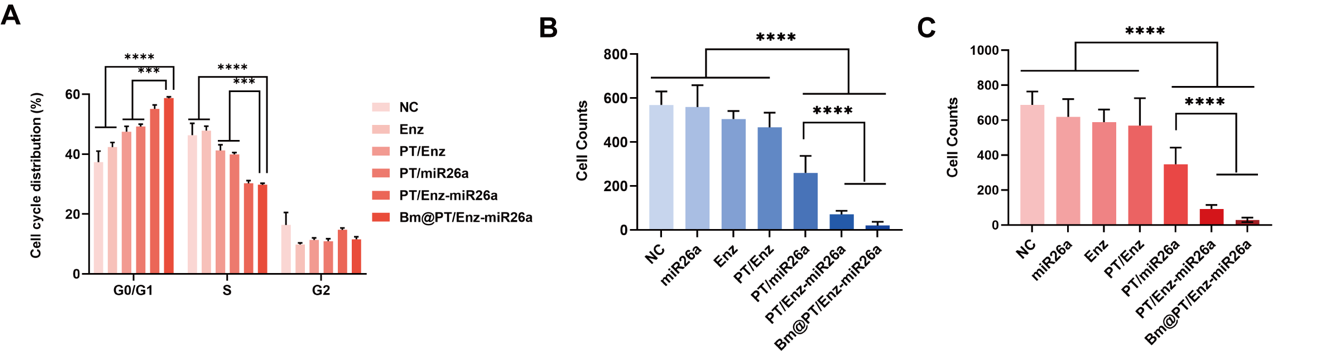
 **Fig. S7. Statistical analysis. (A)** Statistical analysis of cell arrest. n = 3, mean ± SD, ****p* < 0.001, *****p* < 0.0001, one-way ANOVA. **(B-C)** Statistical analysis of each group (anti-migration /anti-invasion), n = 9, mean ± SD, *****p* < 0.0001, one-way ANOVA.

**
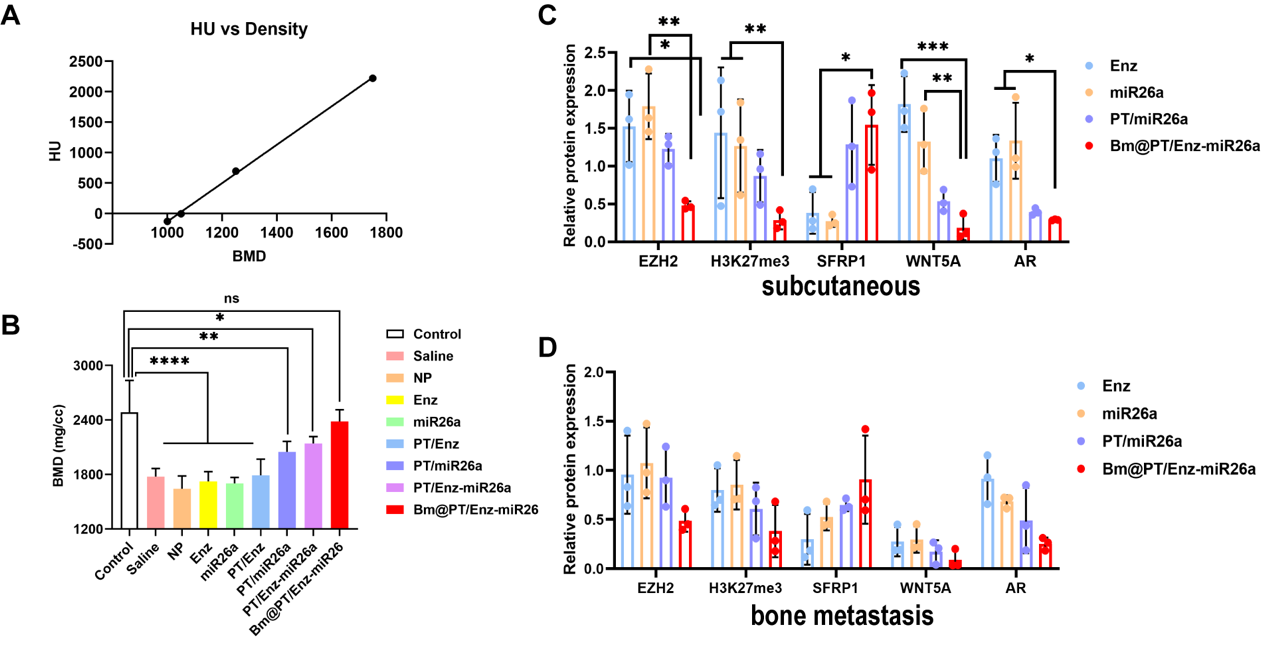
**

**Fig. S8. (A, B)** The BMD values of tumor bearing tibias of each group in BmCRPC mouse model, and tumor-free normal tibias were as control. n = 5, mean ± SD, **p* <0.05, ***p* <0.01, *****p* <0.0001, ns: no significance, one-way ANOVA. **(C, D)** Western blotting results of related proteins in **(C)** subcutaneous CRPC and **(D)** BmCRPC mouse models. n=3, mean ± SD, **p* < 0.05, ***p* < 0.01, ****p* < 0.001, *****p* < 0.0001, one-way ANOVA.

| Group | C4-2B（μg/mL） | C4-2B EnzR（μg/mL） |
| --- | --- | --- |
| NC | NS | NS |
| miR26a | NS | NS |
| Enz | 52.65 | NS |
| PT/Enz | 30.59 | NS |
| PT/miR26a | 0.67 | 1.38 |
| PT/Enz-miR26a | 16.59/0.17 | 32.12/0.32 |
| Bm@PT/Enz-miR26a | 3.63/0.04 | 11.83/0.12 |

**Table S1. IC_50_ values of Enz and/or miR26a in different groups.**
